# Association between metabolic risk factors and hepatocellular carcinoma: a systematic review and meta-analysis of cohort studies

**DOI:** 10.3389/fmed.2026.1865521

**Published:** 2026-07-17

**Authors:** Xiaoli Yang, Peng Fu, Hui Liu

**Affiliations:** 1 Department of Nephrology, Shidong Hospital Affiliated to University of Shanghai for Science and Technology, Yangpu District, Shanghai, China; 2 Key Laboratory of Carcinogenesis and Cancer Invasion of Ministry of Education, Department of Hepatobiliary Surgery and Transplantation, Liver Cancer Institute, Zhongshan Hospital, Fudan University, Shanghai, China

**Keywords:** hepatocellular carcinoma, meta-analysis, metabolic risk factors, metabolic syndrome, survival

## Abstract

**Background:**

Considerable evidence suggests that hepatocellular carcinoma (HCC) is tightly related to metabolic disorders. However, it is not yet clear which metabolic risk factors play a critical role in hepatocarcinogenesis.

**Methods:**

We searched Pubmed, Embase, and Web of Science from inception up to April 29, 2023. The hazard ratio (HR) and the corresponding 95% confidence interval (95%CI) were used to assess the pooled risk.

**Results:**

Seventy-three studies involving more than 45 million participants were included. Among the metabolic risk factors: metabolic syndrome (MetS) (HR = 1.80, 95%CI: 1.44–2.25), diabetes mellitus (DM) (HR = 2.01, 95%CI: 1.79–2.27), body mass index (BMI) ≥ 24 kg/m^2^ (HR = 1.47, 95%CI: 1.35–1.61), waist circumference (WC) (HR = 1.39, 95%CI: 1.26–1.55), waist-to-hip ratio (WHR) (HR = 1.33, 95%CI: 1.16–1.52), waist-to-height ratio (WHtR) (HR = 2.23, 95%CI: 1.42–3.51), reduced triglyceride (TG) (HR = 1.36, 95%CI: 1.09–1.69) and hypertension (HR = 1.43, 95%CI: 1.13–1.81) were positively associated with HCC risk. The disturbances of high-density lipoprotein cholesterol (HDL-C) levels (elevated, HR = 0.88, 95%CI: 0.81–0.96; reduced, HR = 0.96, 95%CI: 0.93–0.99) and elevated low-density lipoprotein cholesterol (LDL-C) (HR = 0.44, 95%CI: 0.32–0.62) were consistently associated with decreased risk of HCC. Nevertheless, elevated total cholesterol (TC) (HR = 0.87, 95%CI: 0.69–1.10) and elevated TG (HR = 0.72, 95%CI: 0.36–1.43) were not significantly associated with HCC risk.

**Conclusion:**

Adverse metabolic risk factors are associated with an increased risk of HCC, which may provide new insights into HCC screening strategies for patients with these risk factors.

**Systematic review registration:**

https://www.crd.york.ac.uk/PROSPERO/view/CRD42023401883.

## 1 Introduction

Hepatocellular carcinoma (HCC) is one of the leading causes of cancer-related death worldwide (1), and considerable progress has been made in the study of the epidemiology, risk factors, and molecular characteristics of HCC in recent decades (2–4). In parallel, although various types of effective treatments, including surgery, radiation, chemotherapy, immune therapy, and targeted therapy, have been developed, the prognosis of HCC is still unsatisfactory. HCC is a complex disease with a multifactorial etiology. Risk factors such as chronic liver disease, obesity, diabetes mellitus (DM), and non-alcoholic fatty liver disease (NAFLD) have been associated with disrupted liver homeostasis, inflammation, oxidative stress, and DNA damage, which may contribute to the development of HCC (5, 6). Although increasing evidence suggests that metabolic diseases, including DM, obesity, dyslipidemia, and hypertension, are associated with an increased risk of HCC, it is not yet clear which metabolic risk factors play a critical role in the occurrence of HCC. Therefore, identifying the risk factors associated with HCC and implementing effective management strategies should be prioritized in the future.

Metabolic dysregulation is one of the hallmarks of cancer, including HCC. For instance, lipid and glucose metabolism are essential to the stability and energy supply of cell membranes, thereby influencing cell growth and proliferation by regulating multiple signaling pathways (7–9). The metabolic syndrome (MetS), including insulin resistance, obesity, dyslipidemia, and hypertension, has now become a truly global problem, with nearly 1/5 of adults affected in most Asian countries (10, 11), and has been reported to be involved in both benign and malignant transformation (12). The nominal definition of MetS is constantly changing, and for this reason, the components of MetS were considered separately (13–15). Lipids and cholesterol mainly include triglycerides (TG), total cholesterol (TC), high-density lipoprotein cholesterol (HDL-C), and low-density lipoprotein cholesterol (LDL-C), and can be exploited by most types of cancer to meet their increased energy demands. Lipid metabolism is altered in the early stage of HCC, and lipid abnormalities play a pivotal role in facilitating the proliferation, invasion, and metastasis of tumor cells while concurrently exerting an influence on the modulation of the tumor microenvironment (16). However, the exact relationship between serum lipid profile and HCC has been unsettled for years. The relationship between DM and HCC is complex, involving various pathophysiological mechanisms such as insulin resistance, inflammation, oxidative stress, and altered lipid metabolism (17, 18). Furthermore, numerous studies have demonstrated the effectiveness of hypoglycemic and lipid-lowering drugs, as well as bariatric surgery, in reducing the risk of HCC (19, 20). Thus, modifying unfavorable metabolic risk factors serves as an effective strategy for HCC prevention. Therefore, elucidating the individual associations between metabolic risk factors and the risk of HCC holds greater value than an overarching narrative, offering enhanced feasibility in clinical practice.

Indeed, clarifying the relationship between metabolic risk factors and HCC is beneficial for our understanding of hepatic carcinogenesis and for the potential development of prevention strategies. Thus, our meta-analysis provides a profound investigation into the correlation between metabolic risk factors and the risk of HCC by extensively incorporating cohort studies.

## 2 Methods

Our systematic reviews and meta-analysis results were designed, organized, and reported in accordance with the priority reporting items for systematic reviews and meta-analyses (PRISMA) (21). Supplementary Table S1 provides PRISMA Checklist. The PROSPERO prospective register of systematic reviews was utilized to register the protocol for this systematic review and meta-analysis (ID: CRD42023401883).

### 2.1 Eligibility criteria

We included prospective and retrospective cohort studies, and the specific inclusion criteria were developed before retrieval. Articles deemed potentially eligible by two authors independently were included if they met all the following criteria: (i) Articles were prospective or retrospective cohort studies. (ii) Articles evaluated the association between metabolic risk factors and the incidence of HCC. (iii) Articles must directly or indirectly provide the hazard ratio (HR) with 95% confidence interval (95%CI) of metabolic risk factors and the occurrence of HCC. This meta-analysis excluded studies that did not meet any of the following inclusion criteria: (i) Review, meta-analysis, letter, and conference abstract. (ii) Case report (*n* < 5). (iii) Articles written in a language other than English. (iv) Studies lacking control or comparison groups. (v) In the case of multiple articles using the same cohort study, only the most recent literature, relevant data, or the study with the largest sample size was considered.

### 2.2 Data sources and search methods

We systematically searched Pubmed, Embase, and Web of Science with the keywords of “HCC,” “metabolic syndrome,” “metabolic risk factors,” “diabetes mellitus,” “body mass index,” “hypertension, “dyslipidemias,” “high-density lipoprotein cholesterol,” and “low-density lipoprotein cholesterol” from inception to April 29, 2023, to find out the correlation between metabolic risk factors and HCC. The detailed search strategy is presented in Supplementary Table S2. There were no restrictions on publication dates for each database, and the search strategy of each database was appropriately adjusted.

### 2.3 Selection process

**Figure 1:**
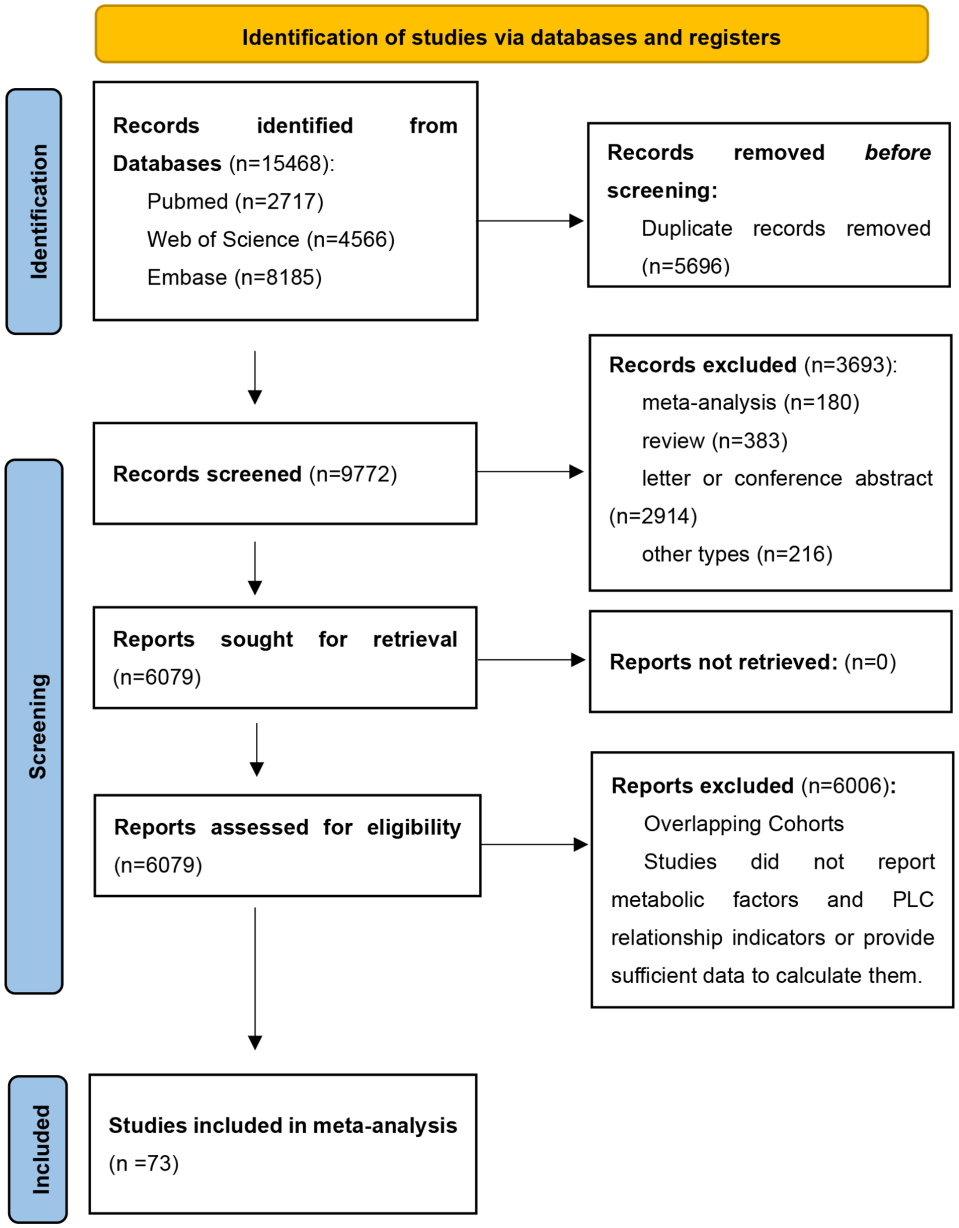
PRISMA flow chart of the study selection process.

Utilizing Endnote 20.5 as the online reference management system, two authors independently reviewed literatures that met the inclusion criteria. An initial exclusion process involved excluding repeated articles using Endnote 20.5 software and preliminarily excluding non-conforming articles by reading their titles and abstracts. In the following step, we read the full text of the included cohort studies to determine if there was a relationship between HCC occurrence and any metabolic risk factor. In the event of any discrepancies, consensus was sought or arbitration was conducted by a third author. The detailed search process and research selection are shown in Figure 1.

### 2.4 Data extraction quality assessment

Separate data extractions were performed by the same authors to form a standardized data table and resolve differences through discussion. We extracted basic characteristics, including overall mean age, gender, year of publication, and countries of origin. In addition, number of patients, median follow-up time, type of metabolic risk, and outcomes (HR with 95%CI). In cases where both univariate and multivariate analysis are available, we analyzed both separately to analyze the impact of internal factors. Regarding HR selection: When a study reported multiple multivariable HR estimates derived from different adjustment models, we selected the most fully adjusted HR (i.e., the model incorporating the greatest number of covariates) as the primary estimate for inclusion in the meta-analysis, as this approach minimizes residual confounding. When adjustment model details were insufficient, consensus was reached by two independent authors through discussion.

We used the Newcastle-Ottawa Scale (NOS) tool to assess the risk of bias in the included studies (22). The assessment was scored on a scale of 0–9, with <5 indicating low quality, 5–7 as moderate quality, and 8–9 as high quality. In the current study, we considered a study that received six or more points as a high-quality study. Supplementary Table S3 provides a detailed.

### 2.5 Statistical analysis

A statistical test for statistical heterogeneity was conducted by using Cochran Q and inconsistency index (I^2^) tests. *I*^2^ < 50% or *p* > 0.1 indicated a lack of heterogeneity, and *p* < 0.1 was considered statistically significant. Meta-analysis was employed to combine the estimates with a fixed effect model utilized in cases where the heterogeneity between the included studies was minimal. Otherwise, we use a random effect model. Funnel plots, Egger’s, and Begg’s tests were used to examine the potential publication bias (23). When there is a significant publication bias, we use the trim and fill method to evaluate the robustness of the results. Statistical analyses were performed using Stata 16.0 statistical software (StataCorp LP, College Station, TX, United States).

## 3 Results

### 3.1 Study selection and characteristics

According to the designated search strategy, 15,468 articles were retrieved from three electronic databases for analysis (2,717 from Pubmed, 4,566 from Web of Science, and 8,185 from Embase). Subsequently, the document management program Endnote 20.5 was employed to check out duplicate literature and eliminate non-original literature. Thereafter, we manually conducted a screening of duplicated literature by comparing critical information, and 785 duplicates were identified and removed from the compilation. During the screening process of the remaining 6,079 articles, a total of 515 articles were retained through reading titles and abstracts of these literatures. Upon obtaining the full text of these 515 articles, a meticulous comparison was conducted, ultimately resulting in the inclusion of 73 articles in our meta-analysis. The details of the flow diagram in this meta-analysis are shown in Figure 1.

After thoroughly reviewing the full text, we have compiled a comprehensive summary of the fundamental characteristics and attributes of the study cohort in Table 1. Our meta-analysis is focused on prospective and retrospective cohort studies, and the level of evidence is superior to that of case–control studies. A total of 73 literatures reported the relationship between metabolic risk factors and HCC. We have extracted four relevant indices of metabolic risk factors, including DM, hypertension, obesity, and dyslipidemia, based on the diverse directions of literature research. The principles outlined in the literature are adhered to when establishing the criteria for DM and hypertension. However, there exists a divergence of opinions regarding obesity-related indicators, such as body mass index (BMI), waist circumference (WC), waist-to-hip ratio (WHR), and waist-to-height ratio (WHtR). To comprehensively investigate the association between dyslipidemia and HCC, four indicators, namely TC, TG, LDL-C, and HDL-C, were extracted. In the literature that did not split the components of metabolic risk factors, the HR of the relationship between MetS and HCC was also uniformly extracted. In the process of data synthesis, it is imperative to consider the variability in the selection of control groups and the corresponding thresholds across different studies.

**Table 1:** Baseline characteristics of studies included in the meta-analysis.

Author	Year	Country	NO. of subjects (Female/Male)	Mean age (years)	Median follow-up time (years)	Metabolic risk factors	Cohort characteristics	NOS score
S. Pelusi et al.	2023	Italy	2,611 (960/1,651)	61.5	2.8	Metabolic syndrome, Diabetes	CHC	8
L. Y. Mak et al.	2023	China	2,330 (1,037/1,293)	54.6	5	Diabetes	CHB	7
F. Kanwal et al.	2023	United States	2,733 (856/1,877)	60	2.85	Diabetes, Hypertension	Cirrhosis	6
T. S. Chang et al.	2022	China	43,545 (25,117/18,428)	56.9	6	Diabetes, Hypertension, Dyslipidemia, BMI	Non-CHB/CHC	8
M. W. Yu et al.	2022	China	1,453	49	19.3	Metabolic syndrome, Diabetes, BMI	CHB	9
Y. Cho et al.	2022	Korea	5,975,308 (2,544,324/3,430,984)	48.6	7.3	Metabolic syndrome, Diabetes, Hypertension, Dyslipidemia	NA	8
R. C. Hsueh et al.	2022	China	2,385 (0/2,385)	43	28.1	BMI	CHB with HBsAg Seroclearance	8
Y. Seko et al.	2022	Japan	1,395 (797/598)	57	4.6	Diabetes, Hypertension	NAFLD	9
B. G. Jun et al.	2022	Korea	14,265,822 (7,695,540/6,570,282)	45	13.7	BMI	NA	8
H. C. Wu et al.	2022	China	18,541 (9,481/9,060)	47.3	13	Diabetes, Hypertension, Dyslipidemia	NA	7
J. A. Rothwell et al.	2022	Britain	366,016 (194,774/171,242)	56.4	7.1	Metabolic syndrome, Diabetes, Hypertension, Dyslipidemia	NA	7
Y. G. Chen et al.	2022	China	13,448 (5,168/8,280)	58.5	7.16	Metabolic syndrome	NAFLD	9
S. Muhimpundu et al.	2021	United States	67,584 (40,979/26,605)	51	10.5	Diabetes	NA	9
Y. Cho et al.	2021	Korea	8,528,790 (3,745,461/4,783,329)	45.4	7.3	Dyslipidemia	NA	8
R. Fan et al.	2021	China	5,754	43.6	4	BMI, WC, WHR, WHtR	CHB	6
D. Ji et al.	2021	China	1,241 (682/559)	50.2	4	Diabetes	CHC-SVR	7
E. Vilar-Gomez et al.	2021	Spain, Australia, Hong Kong, and Cuba	299 (60/139)	57	5.1	Diabetes	NASH with cirrhosis	8
J. N. Benhammou et al.	2021	United States	33,474 (1,071/32,403)	61.1	3	Diabetes	CHC	8
S. Hwang et al.	2021	Korea	9,671,941	46.9	7.3	BMI, WC	NA	8
Y. B. Lee et al.	2021	Korea	317,856 (98,438/219,418)	46	8.5	Metabolic syndrome	CHB	8
C. Rodríguez-Escaja et al.	2021	Spain	982 (209/773)	54	4.1	Diabetes	cirrhosis	9
K. Abe et al.	2020	Japan	188 (98/90)	71	5	Diabetes	CHC with cirrhosis	7
C. T. Lim et al.	2020	Singapore	289 (80/209)	45.2	9.3	Diabetes, Hypertension	NA	6
A. A. Florio et al.	2020	United States and Canada	1,167,244	65	NA	WC, WHR	NA	9
F. Kanwal et al.	2020	United States	271,906 (15,547/256,359)	54.52	9	Diabetes, Hypertension	NAFLD	8
J. D. Yang et al.	2020	United States	354 (209/145)	61.5	3.9	Diabetes, Hypertension	NASH with cirrhosis	7
Y. F. Tan et al.	2019	China	6,564 (2,110/4,454)	45.4	6.3	Metabolic syndrome, Diabetes, Dyslipidemia	CHB	6
Y. C. Shyu et al.	2019	China	5,932 (2,569/3,363)	53.3	11.4	Diabetes	CHB	8
S. Brichler et al.	2019	France	317	NA	5.4	BMI	CHB with cirrhosis	7
K. Björkström et al.	2019	Sweden	406,770 (18,775/219,013)	64.7	7.7	Diabetes	NA	8
K. Kim et al.	2018	Korea	214,167	45.1	8	Diabetes	CHB	8
H. Hagström et al.	2018	Sweden	1,220,261	NA	28.5	BMI	NA	9
A. Wong et al.	2018	Spain	3,503 (1,338/2,165)	54.4	2.3	Metabolic syndrome	CHC with cirrhosis	8
T. C. F. Yip et al.	2018	China	4,568 (1,694/2,874)	56.7	3.4	Diabetes	CHB	7
T. G. Simon et al.	2018	Sweden	171,110 (120,826/50,284)	63.5	14.5	Diabetes	NA	8
S. W. Yi et al.	2018	Korea	504,646	53	10.5	BMI	NA	9
C. F. Huang et al.	2017	China	877 (571/306)	55	8	Diabetes	CHC-SVR	8
M. W. Yu et al.	2017	China	1,690	48.4	19	Metabolic syndrome	CHB	8
B. J. McMahon et al.	2017	United States	1,080	41	10.3	BMI	NA	7
B. Y. Yang et al.	2017	United States	297,928	62	11.9	BMI	NA	9
J. Lee et al.	2016	Korea	102	46.4	3.75	BMI, WC, WHR	CHB	8
P. T. Campbell et al.	2016	United States	1,570,023	NA	NA	BMI, WC	NA	7
M. Hedenstierna et al.	2016	Sweden	180 (56/124)	54	7.8	Diabetes	CHC	8
J. D. Yang et al.	2016	United States	739 (351/388)	58	3.2	Diabetes	CHC	6
V. W. Setiawan et al.	2016	United States	168,476	NA	16.6	BMI	NA	7
H. B. El-Serag et al.	2016	United States	10,738 (506/10,232)	52	2.8	Diabetes	CHC-SVR	8
W. P. Brouwer et al.	2015	Netherlands	531	34	10.1	BMI	CHB	9
J. C. Hsiang et al.	2015	Netherlands	223 (74/149)	51	5	Diabetes	CHB with cirrhosis	7
Y. W. Huang et al.	2015	China	2,187 (1,206/981)	58	5.8	Diabetes	CHC	8
L. Elkrief et al.	2014	France	348 (112/236)	59	4.58	Diabetes	CHC with crirrhosis	6
W. P. Koh et al.	2013	Singapore	61,321 (34,054/27,267)	60	14	Diabetes	NA	9
S. Schlesinger et al.	2013	European countries	359,525	NA	8.6	BMI, WC, WHR, WHtR	NA	8
Y. Arase et al.	2013	Japan	4,302 (1,774/2,528)	52	8.1	Diabetes, Hypertension, Dyslipidemia	CHC	7
S. W. Lai et al.	2012	China	96,745 (42,785/53,960)	56.2	3	Diabetes	NA	8
Y. Kawamura et al.	2012	Japan	6,508 (799/5,709)	49	5.6	Diabetes, Hypertension, Dyslipidemia	NAFLD	6
W. Borena et al.	2012	Norway Australia Sweden	578,700	NA	9.5	BMI	NA	9
L. T. Chao et al.	2011	China	1,142	45	8	Diabetes	CHB	7
C. H. Hung et al.	2011	China	1,470	53	4.4	BMI	CHC	8
R. Loomba et al.	2010	China	2,260	46	18.5	BMI	NA	8
Y. Kawamura et al.	2010	Japan	2,058 (741/1,317)	50	6.7	Diabetes	CHC	8
M. S. Ascha et al.	2010	United States	510	51	3.2	BMI	NA	7
C. S. Wang et al.	2009	China	5,929	NA	8.58	BMI	Viral Hepatitis	8
C. L. Chen et al.	2008	China	23,567	NA	12.5	BMI, WC	CHB/CHC	6
T. Ohki et al.	2008	Japan	1,431	60	6.1	BMI	CHC	8
B. J. Veldt et al.	2008	Europe and Canda	541 (171/370)	50	4	Diabetes	CHC with cirrhotic	8
Y. Torisu et al.	2007	Japan	47 (0/47)	57	6.8	Diabetes	cirrhosis	6
G. N. Ioannou et al.	2007	United States	2,126 (57/2,069)	53	3.6	Diabetes	Cirrhosis	9
G. N’Kontchou et al.	2006	France	771 (249/522)	61	4.2	Diabetes	Cirrhosis	8
M. S. Lai et al.	2006	China	54,916 (30,468/20,848)	NA	2.7	Dyslipidemia	NA	8
S. W. Oh et al.	2005	Korea	781,283	NA	9.9	BMI	NA	8
K. Ohata et al.	2003	Japan	161	53	6.4	BMI	CHC	8
S. Nair et al.	2002	United States	19,271	50	NA	BMI	NA	9
A. Wolk et al.	2001	China	4,841	46	10.3	BMI	NA	7

NOS, Newcastle-Ottawa Scale; CHC, chronic hepatitis C; CHB, chronic hepatitis B; BMI, body mass index; HBsAg, hepatitis B surface antigen; NAFLD, nonalcoholic fatty liver disease; WC, waist circumference; WHR, waist-to-hip ratio; WHtR, waist-to-height ratio; NASH, nonalcoholic steatohepatitis; SVR, sustained virologic response.

### 3.2 MetS and the risk of HCC

**Figure 2:**
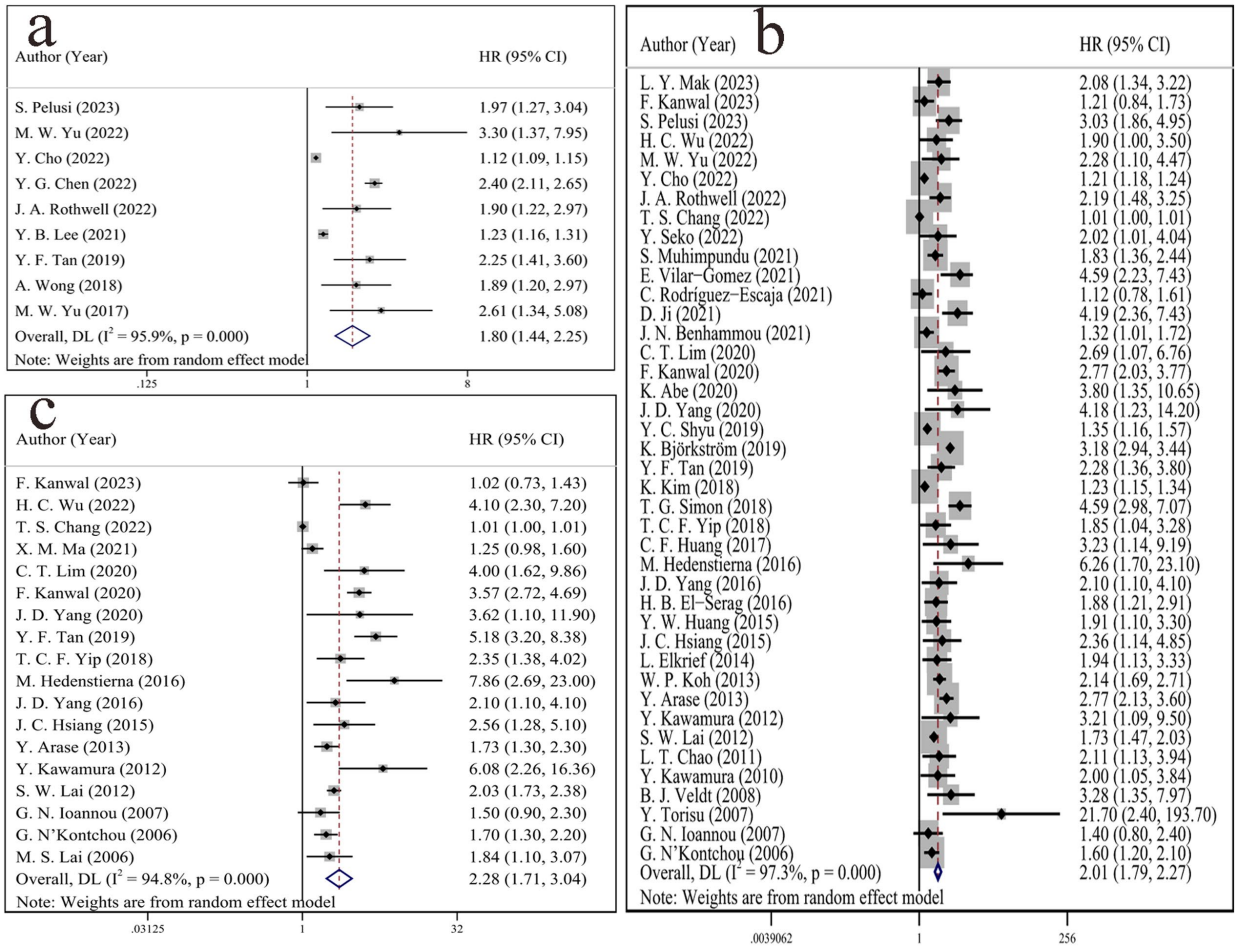
Forest plots of effect between HCC occurrence and metabolic risk factors. **(a)** HR of MetS; **(b)** HR of DM with covariate adjustment; **(c)** HR of DM without covariate adjustment.

Our study encompassed a total of 9 articles investigating the correlation between MetS and HCC. Pooled results from 9 independent articles (24–32) showed that MetS significantly increased the risk of HCC (HR = 1.80, 95%CI: 1.44–2.25, Figure 2a). The outcomes of the heterogeneity analysis indicated substantial variation among the incorporated literature (*I*^2^ = 95.9%, *p* < 0.001). Consequently, we conducted a subgroup analysis on four distinct categories, namely cohort population characteristics, number of subjects, follow-up duration, and countries of origin (Supplementary Figure S2). There was a decrease in heterogeneity in each analysis, which was more significant in the subgroups of population characteristics and number of participant. We observed differences between the subgroup analysis results and the original pooled results regarding the characteristics of the population. The findings revealed a strong association between MetS and the risk of HCC for chronic hepatitis B (CHB) patients (HR = 2.02, 95%CI: 1.20–3.41, *p* = 0.001) or chronic hepatitis C (CHC) patients (HR = 1.93, 95%CI: 1.41–2.64, *p* < 0.05), while in the non-specific population cohort, no significant association was observed between MetS and the risk of HCC (HR = 1.39, 95%CI: 0.84–2.31, *p* > 0.05, Supplementary Figure S1a). In addition, the association between MetS and the risk of HCC was found to be less pronounced in subgroups comprising more than 10,000 participants (HR = 1.54, 95%CI: 1.16–2.05, *p* < 0.001, Supplementary Figure S1b), and a more significant difference was observed in the pooled HR upon grouping based on median follow-up time whether exceeding 10 years (follow up time < 10 years: HR = 1.69, 95%CI: 1.34–2.14, *p* < 0.001; follow up time ≥ 10 years: HR = 2.84, 95%CI: 1.67–4.84, *p* = 0.677, Supplementary Figure S1c). The subgroup hailing from China (HR = 2.41, 95%CI: 2.16–2.68, *p* = 0.889) exhibited a greater contribution to the incidence of HCC in comparison to subgroups from other nations (HR = 1.30, 95%CI: 1.14–1.48, *p* < 0.001, Supplementary Figure S1d).

For the pooled results of MetS and HCC risk, the funnel plot was used to test for publication bias. Due to the asymmetry of the visual funnel plot (Supplementary Figure S2a), we used egge’s test (*p* = 0.047, Supplementary Figure S2b) instead of begg’s test (*p* = 0.348, Supplementary Figure S2c) to confirm that there was indeed publication bias. After supplementing the data of 6 virtual studies with the trim and fill method, the modified effect size did deviate from the preceding estimate (modified HR = 1.233, 95%CI = 0.986–1.542, Supplementary Figure S2d). This suggests that the relationship between MetS and HCC onset needs to be interpreted with caution.

### 3.3 DM and the risk of HCC

For DM, this meta-analysis encompassed 41 articles (24–27, 30, 33–68) following up with a staggering 7,790,213 individuals to investigate the correlation between DM and HCC risk. We extracted the results of univariate analysis and multivariate analysis and combined them using a random effect model, respectively. The findings revealed that patients with DM exhibited a greater susceptibility to HCC in comparison to those with normal blood glucose levels (adjusted HR = 2.01, 95%CI: 1.79–2.27, *p* < 0.001, Figure 2b; unadjusted HR = 2.28, 95%CI: 1.71–3.04, *p* < 0.001, Figure 2c), but the results demonstrated significant heterogeneity (adjusted HR, *I*^2^ = 97.3%, *p* < 0.001; unadjusted HR, *I*^2^ = 94.8%, *p* < 0.001). Given that multivariate HR incorporates numerous variables and possesses greater applicability and universality, we solely undertake subgroup analysis on multivariate HR to examine potential sources of heterogeneity.

Subgroup analysis was conducted based on the fundamental characteristics of the cohort (Supplementary Figure S3a) and the source region of the cohort (Supplementary Figure S3b). Within the fundamental characteristics of the cohort, those with hepatitis virus infection complicated by cirrhosis were categorized as the virus infection group, while those with cirrhosis of unspecified etiology were categorized under the unspecified cause of cirrhosis group. The synthetic effect indicators revealed that the correlation between DM and HCC was more pronounced in certain subgroups, including patients with CHC (HR = 2.26, 95%CI: 1.73–2.97, *p* = 0.007), NAFLD (HR = 2.63, 95%CI: 1.98–3.49, *p* = 0.415), NASH with cirrhosis (HR = 4.51, 95%CI: 2.63–7.73, *p* = 0.893), and patients with CHC-SVR (HR = 2.81, 95%CI: 1.58–5.00, *p* = 0.084), but not in unspecified cause of cirrhosis group (HR = 1.39, 95%CI: 1.03–1.88, *p* = 0.063). After restricting the cohort area, it was determined that the risk of HCC in diabetic populations is greater in European (HR = 2.41, 95%CI: 1.70–3.40, *p* < 0.001) countries compared to Asian (HR = 1.72, 95%CI: 1.53–1.94, *p* < 0.001) and American countries (HR = 1.77, 95%CI: 1.38–2.26, *p* = 0.006). In terms of heterogeneity, a decrease was observed across all subgroups except for the non-specific population subgroup (*I*^2^ = 98.5%, *p* < 0.001, Supplementary Figure S4a).

The presence of serious publication bias was detected through the asymmetry of the funnel plot, which was further confirmed by the application of egger’ test (*p* < 0.001, Supplementary Figure S4b) and begg’ tests (*p* = 0.017, Supplementary Figure S4c). To address this issue, we further undertook analysis using the trim-and-fill method. Despite minor alterations to the revised effect indicator in comparison to the original data, the statistical significance of the results remained intact, indicating greater robustness (modified HR = 1.204, 95%CI = 1.068–1.356, Supplementary Figure S4d).

### 3.4 Obesity and the risk of HCC

This meta-analysis incorporated 28 articles (25, 35, 69–87) to assess the correlation between obesity and HCC risk. We assessed the following four obesity indices: BMI, WC, WHR, and WHtR. Among these articles, 28 presented relevant data pertaining to the association between BMI and HCC risk. According to the definition provided by the World Health Organization (WHO) regarding obesity and overweight, overweight is defined as a BMI equal to or greater than 25 kg/m^2^, while obesity is defined as a BMI equal to or greater than 30 kg/m^2^. However, considering regional variations and racial differences, the WHO has set a lower threshold for overweight, specifically for Asian populations, defining overweight as having a BMI equal to or greater than 24 kg/m^2^. Not all the identified studies compared the differences between the three groups for outcomes and the cut-off values of BMI varied, so we defined BMI ≥ 24 kg/m^2^ as general adiposity in this meta-analysis. For ease of comparison, we categorized participants into the normal weight, overweight, and obese groups based on the threshold values identified in various studies. But in subgroup analysis, we will adhere to the WHO’s definitions of obesity and overweight while considering the variations in thresholds among the included references, ensuring that the differences in criteria for classification are carefully addressed.

**Figure 3:**
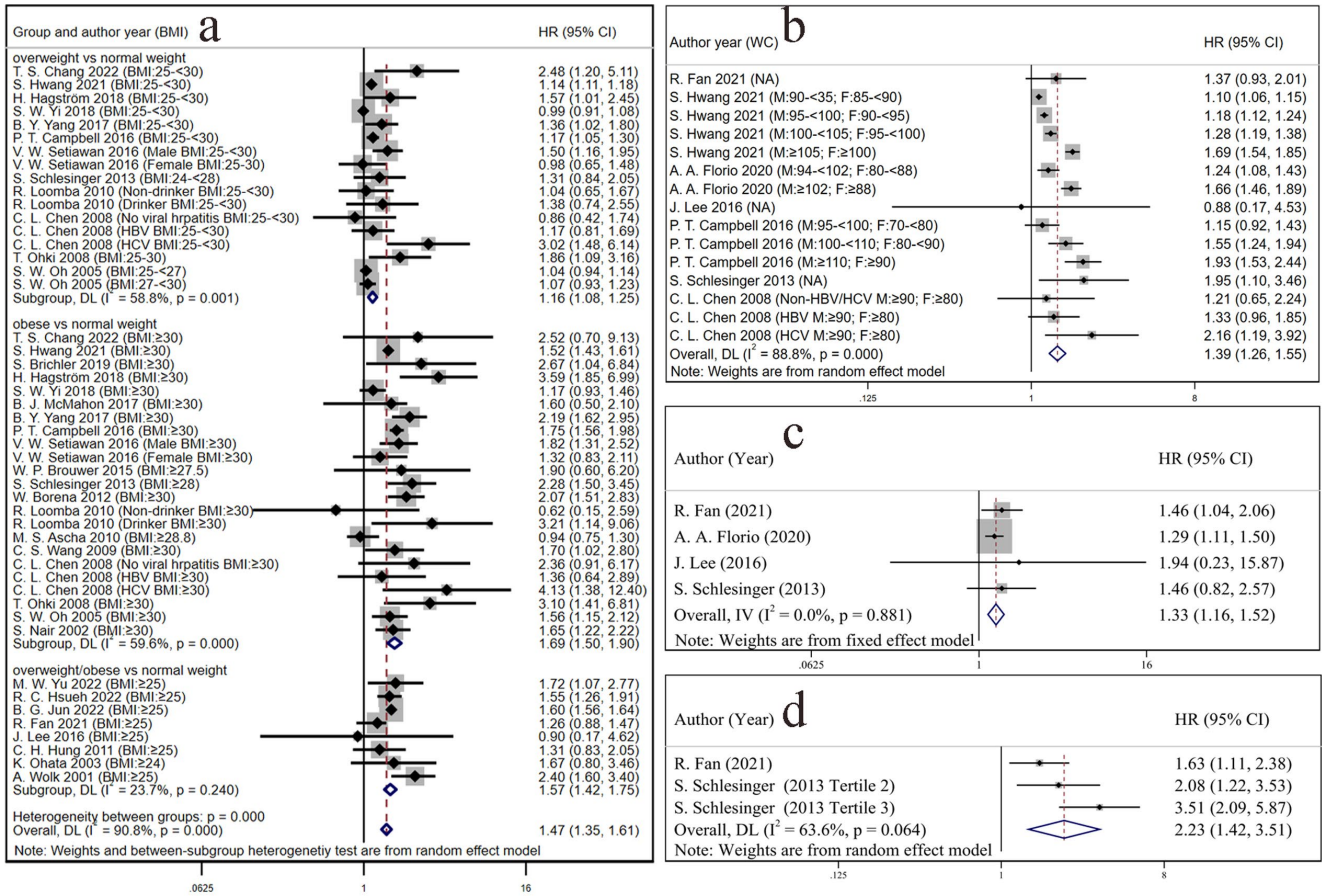
Forest plots of effect between HCC occurrence and obesity. **(a)** BMI; **(b)** WC; **(c)** WHR; **(d)** WHtR.

The pooled HR for the risk of HCC in patients with general adiposity (BMI ≥ 24 kg/m^2^) was 1.47 (95%CI: 1.35–1.61, *p* < 0.001, Figure 3a) compared to those normal weight categories. Likewise, significant heterogeneity was found in the analysis (*I*^2^ = 90.8%, *p* < 0.001). Subgroup analysis was performed according to BMI range, personnel characteristics, and continent. Compared with the BMI < 25 kg/m^2^ group, the HR of HCC in the BMI ≥ 25 kg/m^2^ group, BMI of 25–30 kg/m^2^ group, and BMI ≥ 30 kg/m^2^ group were 1.57 (95%CI: 1.40–1.77, *p* = 0.165), 1.21 (95%CI: 1.10–1.34, *p* = 0.001), and 1.72 (95%CI: 1.54–1.93, *p* = 0.007, Supplementary Figure S5a). Based on cohort characteristics, the pooled HR for the incident risk of HCC in patients with CHB, CHC, and CHB/CHC were 1.48 (95%CI: 1.27–1.72, *p* = 0.533), 1.73 (95%CI: 1.25–2.39, *p* = 0.305), and 1.68 (95%CI: 1.07–2.63, *p* = 0.035, Supplementary Figure S5b). Regarding regional variation, we conducted intercontinental subgroup analysis and found that there is no difference between the pooled HR of these subgroups [Asia (HR = 1.42; 95%CI = 1.27–1.58, *p* < 0.001); America (HR = 1.44; 95%CI = 1.22–1.69, *p* < 0.001); Europe, HR = 1.97 (95%CI = 1.45–2.67, *p* < 0.05), Supplementary Figure S5c]. The funnel plot with good symmetry showed that there was no obvious publication bias (Supplementary Figure S6a), which was reinforced by the egger’ test (*p* = 0.567, Supplementary Figure S6b) and begg’ tests (*p* = 0.230, Supplementary Figure S6c).

As for central adiposity, we explored the association between HCC risk and WC, WHR, and WHtR. The pooled HR of WC and HCC risk was 1.39 (95%CI: 1.26–1.55, *p* < 0.001, Figure 3b), showing significant heterogeneity (*I*^2^ = 88.8%, *p* < 0.001). Only four articles analyzed the relationship between WHR and HCC risk, and two articles discussed the relationship between WHtR and HCC risk. The results reflected that elevated WHR and WHtR were associated with an increased risk of HCC (WHR: HR = 1.33, 95%CI = 1.16–1.52, *p* = 0.881; WHtR: HR = 2.23, 95%CI = 1.42–3.51, *p* = 0.064, Figures 3c,d). No publication bias was detected in the analysis of WC, WHR, or WHtR by either test. The specific results are presented in Supplementary Table S4.

### 3.5 Dyslipidemia and the risk of HCC

**Figure 4:**
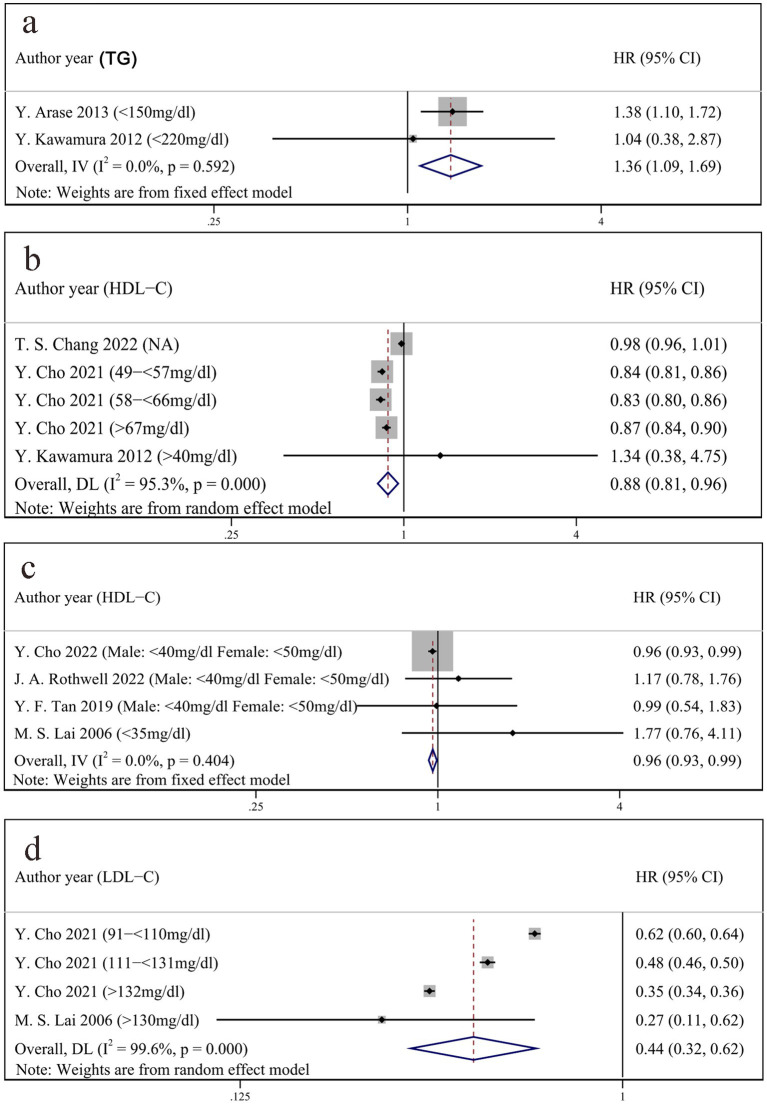
Forest plots of effect between HCC occurrence and dyslipidemia. **(a)** TG; **(b)** TC; **(c)** HDL; **(d)** LDL.

Nine studies (26, 27, 30, 35, 37, 60, 62, 88, 89) comprising 15,004,490 participants were conducted to investigate the correlation between dyslipidemia and HCC risk. However, since the definition of dyslipidemia is not uniform in certain literature sources, we excluded them to ensure data accuracy. The threshold values of TC, TG, HDL-C, and LDL-C, and the effect indicator (HR and 95%CI) of the relationship between abnormal levels and HCC were extracted from nine literatures. The results showed that reduced TG levels were associated with an elevated risk of HCC (HR = 1.36, 95%CI: 1.09–1.69, Figure 4a). There was significant heterogeneity in the study (*I*^2^ = 92.1%, *p* < 0.001). It is noteworthy that increase (HR = 0.88, 95%CI: 0.81–0.96, *I*^2^ = 95.3%, *p* < 0.001, Figure 4b) or decrease (HR = 0.96, 95%CI: 0.93–0.99, *I*^2^ = 0.00%, *p* = 0.404, Figure 4c) of HDL-C is negatively correlated with the occurrence of HCC, and the high level of LDL-C (HR = 0.44, 95%CI: 0.32–0.62, *I*^2^ = 99.6%, *p* < 0.001, Figure 4d) is also exhibiting an inverse correlation with the risk of HCC. However, it may be limited to the small number of related studies, when focusing on the relationship between the risk of HCC and elevated TC (HR = 0.87, 95%CI: 0.69–1.10, *I*^2^ = 92.1%, *p* < 0.001, Supplementary Figure S7a) or TG (HR = 0.72, 95%CI: 0.36–1.43, *I*^2^ = 93.9%, *p* < 0.001, Supplementary Figure S7b) levels, no statistically significant correlation was observed. No publication bias was detected in these groups, whether using the funnel plot or egger’s test and begg’s test. The specific results are presented in Supplementary Table S4.

### 3.6 Hypertension and the risk of HCC

**Figure 5:**
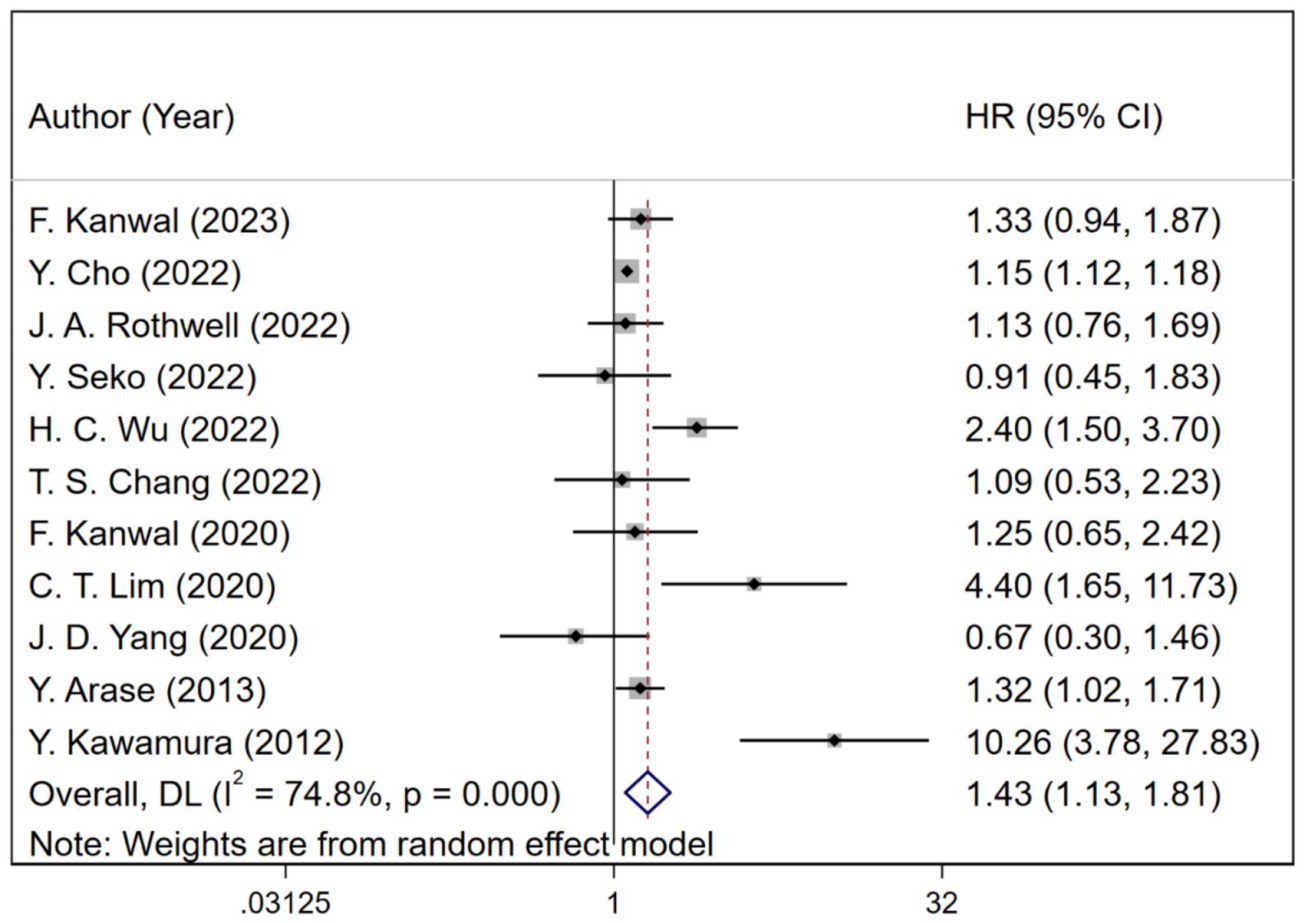
Forest plots of effect between HCC occurrence and hypertension.

Eleven studies (26, 27, 34–37, 44–46, 60, 62) with 6,690,897 participants evaluated the relationship between hypertension and the risk of HCC. Overall, participants with hypertension experienced an increased risk of developing HCC compared to those with normotensive (HR = 1.43, 95%CI: 1.13–1.81, *p* < 0.001). There was moderate heterogeneity across the studies (*I*^2^ = 74.8%, *p* < 0.001, Figure 5). Subgroup analysis was performed primarily based on personnel characteristics, median follow-up time, number of participants, and continent. The subgroup analysis revealed that the cohort of the non-specific population yielded favorable outcomes (HR = 1.65, 95%CI: 1.05–2.58). However, there were no significant associations between hypertension and the risk of HCC in the subgroups comprising individuals with cirrhosis (HR = 1.04, 95%CI: 0.55–1.98) or NAFLD (HR = 2.15, 95%CI: 0.60–7.68, Supplementary Figure S8a). Subsequently, after grouping the total number of participants, we found the pooled HR becomes more meaningful when considering combinations with fewer than 10,000 participants (HR = 1.69, 95%CI: 1.01–2.81, Supplementary Figure S8b). In the subgroup with a median follow-up time of fewer than 5 years, no significant causal relationship was found between hypertension and the risk of HCC (HR = 1.06, 95%CI: 0.71–1.57), but this relationship was relatively evident in the subgroup with a median follow-up time exceeding 5 years (HR = 1.64, 95%CI: 1.21–2.23, Supplementary Figure S8c). These findings suggest that an adequate follow-up time may be pivotal in minimizing testing errors. By splitting the pooled HR in accordance with continent, the results announced positive results in the cohort selected in Asia (HR = 1.74, 95%CI: 1.20–2.51), while in the America group, there is no association between hypertension and HCC (HR = 1.17, 95%CI: 0.83–1.65). The funnel plot shows symmetry, thereby suggesting no publication bias (Supplementary Figure S9a), which was confirmed by egger’s test (*p* = 0.116, Supplementary Figure S9b) and begg’s test (*p* = 0.640, Supplementary Figure S9c).

## 4 Discussion

In this meta-analysis, we pooled data from 73 studies with more than 45 million participants to provide a comprehensive and systematic review of the association between metabolic risk factors and HCC risk. There is a growing awareness of the importance of managing multiple risk factors that contribute to the heightened risk of HCC. It is also recognized that certain drugs for the treatment of metabolic syndrome may inhibit these risk factors and decrease the risk of HCC. Based on the research of Kassi et al. (90), we conducted a meta-analysis mainly focusing on four components of the metabolic syndrome: DM, obesity, dyslipidemia, and hypertension, taking into full consideration the differences in different definitions. We extracted four obesity indexes, namely BMI, WC, WHR, and WHtR, and four dyslipidemia indexes, namely TC, TG, HDL-C, and LDL-C, to elucidate the primary indicators influencing the onset of HCC. According to the study of Eckel et al. (91), MetS is a common metabolic disorder caused by the increasing incidence of obesity, and they point out that this concept will have different interpretations and be widely accepted in the future. We aim to investigate which metabolic risk factors are associated with the risk of HCC to provide guidance for the clinical prevention of HCC.

As an important metabolic risk factor, DM has been favored by most researchers because of its involvement in the pathogenesis and prognosis of various cancers, including HCC. In past meta-analyses, diabetic patients were found to have a greater risk of HCC than people with normal blood glucose (92). Although previous meta-analyses have indicated an increased risk of HCC in diabetic patients, our study is the most comprehensive and up-to-date investigation, employing extensive subgroup analyses to explore heterogeneity. The essence of the paper is that the new combined results are consistent with the original aggregated results, even after including the updated data in recent years. In addition, the pooled HR values obtained by univariate analysis and multivariate analysis showed that DM was significantly associated with the incidence of HCC. Through subgroup analysis, we identified the possible sources of heterogeneity in the study, namely the characteristics of the participating population and the source region of the cohort. It is worth noting that in several articles reporting negative results, the characteristics of the participants belonged to the cirrhosis group (34, 42, 67). This suggests that the relationship between DM and HCC in patients with cirrhosis requires careful interpretation. A longitudinal study conducted by Simon et al. (51), spanning over 26 years, has demonstrated an elevated risk of HCC in individuals with DM, wherein this risk positively correlates with the duration of DM and the number of comorbid metabolic components. While a substantial body of evidence suggests an augmented risk of HCC in the presence of DM, the intricate mechanisms underlying this association remain enigmatic. Several pathophysiological mechanisms have been proposed to explain the observed association between DM and HCC risk, including insulin resistance, dysregulation of glucose and lipid metabolism, and aberrant release of inflammatory mediators, which may collectively influence the tumor immune microenvironment. However, causal inference cannot be drawn from the observational studies included in this meta-analysis (93, 94). Some evidence suggests that pharmacological management of DM, such as metformin, may confer additional protective effects against HCC risk, though this was not directly assessed in the present meta-analysis and warrants dedicated investigation (95, 96). Therefore, it is speculated that metformin has been associated with a reduced risk of the occurrence of HCC by improving the microenvironment (97).

Another notable finding of our study is that adiposity-related measures were significantly associated with the risk of HCC. Adiposity-related measures primarily encompass the following aspects: BMI, WC WHR, and WHtR. In addition, the pooled HR was significantly higher for patients with BMI > 30 kg/m^2^ compared to those with BMI ranging from 25 to <30 kg/m^2^. Due to the limited number of studies reporting central obesity indicators, we used WC as the primary central obesity indicator. In terms of comorbidities, we found a synergistic effect between chronic viral hepatitis and general obesity, as well as chronic viral hepatitis and central obesity, in increasing the risk of HCC, suggesting that excess body fat may be associated with accelerated the process of HCC development in patients with viral hepatitis. BMI is anthropometrically limited because it does not assess individual components of body weight, such as body fat distribution or muscle volume (98). Regional body fat distribution plays a crucial role in patients with MetS (99). According to previous studies, visceral fat accumulation, but not BMI, is closely related to the prognosis of HCC (100). Studies have shown that patients with cirrhosis who have more visceral fat accumulation are more likely to develop liver cancer than those without visceral fat accumulation (101). The difference between the study and previous studies may be attributed to differences between the races, with Japanese people accumulating fat at a higher rate than Caucasians and Africans (102). A meta-analysis by Yan et al.’s compared BMI with WC by measuring the incidence density of liver cancer among obese individuals and characterizing them accordingly. There was a greater incidence of liver cancer among those with excess WC than those with excess BMI, suggesting that visceral adiposity may play a greater role, and WC may have more potential for predicting liver cancer than BMI (103). Another study conducted by Yamagishi et al. unveiled that obesity may be associated with the development of HCC possibly through its impact on the tumor microenvironment, leading to the suppression of anti-tumor immunity (104). In addition, it has been demonstrated in studies that tumor tissue from obese patients harbors a high burden of senescent cells, suggesting that obesity is associated with tumor growth promotion through the induction of cellular senescence (105).

Although dyslipidemia is an established risk factor for cardiovascular disease and several cancers, the relationship between dyslipidemia and HCC remains unclear (106, 107). The current study found that reduced TG was associated with an increased risk of HCC, while elevated TC showed no significant association. Furthermore, we observed an inverse association between HCC risk and dysfunctional HDL-C. It should be noted that elevated TC and TG were not associated with HCC. In contrast, a prospective study based in Taiwan showed that TC > 150 mg/dL was associated with a decreased risk of HCC (35). Results from another Nationwide Population-Based Study in South Korea showed that low lipid levels may be an independent risk factor and preclinical marker for HCC. After adjusting for BMI, hypertension, and DM, elevated levels of TC, TG, HDL, and LDL-C were found to be negatively correlated with the development of HCC (88). This reverse causation framework is supported by evidence that plasma levels of TC, TG, LDL-C, HDL-C, and apolipoproteins are broadly decreased in HCC patients, reflecting the degree of hepatocellular functional impairment rather than a primary lipid disorder (108). Consistent with this, a large nationwide cohort study of over 8.5 million individuals confirmed that low serum lipid levels served as an independent risk factor and preclinical marker for HCC (88). Furthermore, a Mendelian randomization analysis provided genetic-level evidence that HCC itself exerts a negative causal effect on circulating LDL-C levels, further supporting the presence of reverse causation in the observed lipid-HCC associations (109). The unexpected finding that both elevated and reduced HDL-C levels were inversely associated with HCC risk may be explained by the dual role of HDL-C dysfunction: structurally abnormal HDL-C particles produced in the setting of chronic liver disease or metabolic dysregulation may lose their atheroprotective and anti-tumor properties regardless of their absolute concentration, thereby potentially reflecting a state of dysfunctional lipid metabolism rather than a straightforward quantitative association. This suggests that the risk factors for HCC vary with the cause of the disease. A study conducted by Tan et al. (30) found no association between dyslipidemia and HCC development in CHB patients, suggesting that the presence of factors such as CHB that may affect the blood lipid level of patients may mask the association between dyslipidemia itself and the development of HCC. Despite this, there may be some intrinsic relationship between dyslipidemia and HCC, which may result in large differences in results. Therefore, more intensive clinical follow-up is expected to explain the source of this difference. Emerging evidence also suggests that lipoprotein(a), a recently recognized lipid biomarker, may serve as a prognostic marker in HCC, with lower lipoprotein(a) levels observed in HCC patients compared to healthy controls, potentially reflecting impaired hepatic synthetic function. These observations collectively underscore the need for prospective studies with serial lipid measurements and careful adjustment for underlying liver function when investigating lipid-HCC associations (110).

Current research on hypertension and cancer focuses on the hypertensive burden in cancer patients and the hypertension caused by antineoplastic drugs (111, 112). To our knowledge, this meta-analysis represents a pioneering endeavor in elucidating the association between hypertension and the risk of HCC, and the summary results showed that participants with hypertension were more likely to develop HCC, whereas in subgroup analysis, we found no significant association between hypertension and HCC in those with cirrhosis or NAFLD. This means that hypertension may not be an independent risk factor for HCC but is often influenced by comorbidities. We speculate that one of the reasons for this result is that hypertension and HCC have many overlapping risk factors, and there may be some common pathophysiological signaling pathways (113, 114). Additionally, we found that a long follow-up period of more than 5 years was required to detect the cumulative effect of hypertension on HCC development. The results remain relatively robust, as the number of patients does not significantly change the results.

Several outcomes in this meta-analysis demonstrated considerable between-study heterogeneity (DM: *I*^2^ = 97.3%; MetS: *I*^2^ = 95.9%; BMI: *I*^2^ = 90.8%; reduced TG: *I*^2^ = 92.1%). Although subgroup analyses identified population characteristics, geographic region, follow-up duration, and sample size as potential contributors, substantial residual heterogeneity persisted, likely reflecting unmeasured differences in covariate adjustment, exposure definitions, and underlying liver disease severity. The reported HRs should therefore be interpreted as approximate estimates of the direction and magnitude of association rather than precise effect sizes generalizable across clinical settings. To summarize, our research has provided comprehensive insights into the metabolic factors associated with HCC. This significant achievement bears significant clinical and public health implications, enhancing the response strategies toward the prevention of HCC. On the one hand, the types of studies we included were all cohort studies, which ensured that the exposure factors appeared before the outcome and improved the persuasiveness of the conclusions. On the other hand, we pooled the HR of each included study to assess the strength of the association, performed more detailed subgroup analyses to explore comorbidity effects, and discussed possible biological mechanisms. In addition, we also added evidence, including the potential contribution of metformin to HCC prevention by improving the tumor immune microenvironment, and the promotion of tumor growth by obesity through suppression of anti-tumor immune responses.

It should be acknowledged that our systematic review and meta-analysis have some limitations, although we have tried our best to control for errors. First, our included studies primarily consisted of retrospective cohort studies. Second, the study population included patients with various conditions, such as CHB, CHC, NASH, etc., and some of them were undergoing antiviral therapy, which inevitably made the pooled results show significant heterogeneity. Third, among the included studies, there are differences in the cut-off values of several indicators, especially obesity measures and serum lipid levels. However, we conducted a relevant subgroup analysis and classified the values accordingly. The results of each subgroup analysis were consistent with the combined results. Fourth, evidence of significant publication bias was observed in both the overall analysis of MetS and the analysis of DM, with some evidence of negative or neutral results missing. After adjusting HR using the trim and fill method, the association between MetS and HCC was significantly weakened, while the association between DM and HCC did not change much. Fifth, there are too few studies on dyslipidemia and HCC occurrence, and it can be noted that the results of different studies differ greatly, which may be related to the different characteristics of the included population. However, the subgroup analysis was unable to carry out due to limited studies, and we could not further analyze the intrinsic relationship between HCC occurrence and lipid indexes in different populations. Sixth, the majority of the studies in our meta-analysis were not conducted in African populations, so caution is advised when generalizing our findings to African populations. Seventh, substantial residual heterogeneity persisted across most analyses despite subgroup stratification (e.g., DM: *I*^2^ = 97.3%; MetS: *I*^2^ = 95.9%), limiting the precision of pooled estimates and their generalizability to specific patient populations. Meta-regression was not feasible given the limited number of studies in certain subgroups; future analyses with larger study pools are encouraged to formally quantify sources of between-study variance. Eighth, emerging biomarkers such as HOMA-IR, which may more precisely capture insulin resistance beyond a binary DM diagnosis, were not captured in our quantitative synthesis; future prospective studies should consider their inclusion. All included studies were observational cohort studies; despite multivariable adjustment, residual confounding from unmeasured variables, such as alcohol consumption, fibrosis stage, antiviral therapy use, and dietary factors, cannot be excluded. The findings of this meta-analysis should therefore be interpreted as reflecting statistical associations rather than causal relationships. Future studies with larger numbers of eligible studies should consider meta-regression to more rigorously explore the sources of between-study heterogeneity.

## 5 Conclusion

In summary, adverse metabolic risk factors are associated with an increased risk of HCC, including DM, obesity (BMI, WC, WHR, WHtR), dyslipidemia (TC, TG, HDL-C, LDL-C), and hypertension. It should be noted that the association between overall MetS and HCC risk became non-significant after adjustment for publication bias, indicating that this specific association should be interpreted with considerable caution pending further confirmation from additional high-quality studies.

## Data Availability

The original contributions presented in the study are included in the article/Supplementary material, further inquiries can be directed to the corresponding author.

## Glossary

HCC: Hepatocellular carcinoma
HR: Hazard ratio
95%CI: 95% confidence interval
MetS: Metabolic syndrome
DM: Diabetes mellitus
BMI: Body mass index
WC: Waist circumference
WHR: Waist-to-hip ratio
WHtR: Waist-to-height ratio
TG: Triglyceride
TC: Total cholesterol
HDL-C: High-density lipoprotein cholesterol
LDL-C: Low-density lipoprotein cholesterol
NAFLD: Nonalcoholic fatty liver disease
NOS: Newcastle-Ottawa scale
CHC: Chronic hepatitis C
CHB: Chronic hepatitis B
NASH: Non-alcoholic steatohepatitis
SVR: Sustained virologic response
WHO: World health organization
