# Supplementary material for: Association between metabolic risk factors and hepatocellular carcinoma: a systematic review and meta-analysis of cohort studies
Source: Front Med (Lausanne). 2026 Jul 17;13:1865521. doi: 10.3389/fmed.2026.1865521 (PMC13424207; doi:10.3389/fmed.2026.1865521)
Supplement: Supplementary file 1 [file Supplementary_file_1.docx]

**Association between metabolic risk factors and** **hepatocellular carcinoma: A systematic review and meta-analysis of cohort studies**

Xiaoli Yang1,2, Peng Fu1,2, Hui Liu3
1Department of Nephrology, Shidong Hospital, Yangpu District, Shanghai 200438, China.
2Department of Nephrology, Shidong Hospital Affiliated to University of Shanghai for Science and Technology Shanghai 200438, China.
3Department of Hepatobiliary Surgery and Transplantation, Liver Cancer Institute, Key Laboratory of Carcinogenesis and Cancer Invasion of Ministry of Education, Zhongshan Hospital, Fudan University, China.
.
Corresponding author: Hui Liu
Address: 180Fenglin Road, Shanghai, 200032, China
Tel & Fax: +86-178-12184089.
Email: Liuhuidoct@163.com

**Catalogue**

[Supplementary Figures 2-](#_Toc115779687)10

[Supplementary Tables 11-](#_Toc115779688)25


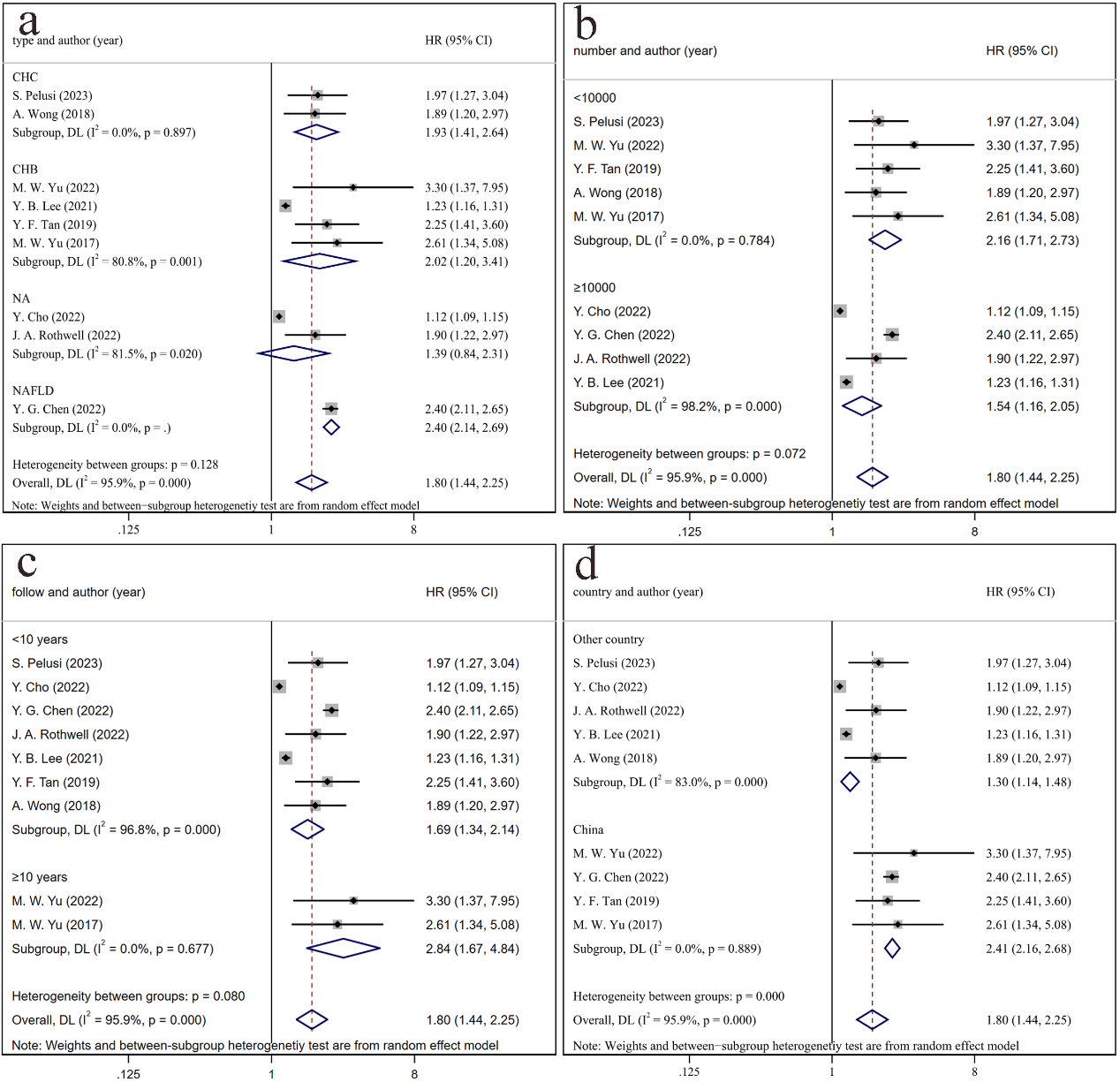


**Figure S1** **Forest plots of subgroup showing the association between MetS and HCC risk:** (a) cohort characteristics, (b) number of subjects, (c) media follow-up time, (d) cohort source region. For the black diamond, its position represents the size of the HR studied, and the size of the square in grey surrounding the diamond represents the weight in the analysis. The length of the transverse line represents the corresponding 95% CI. **Abbreviation**: MetS, metabolic syndrome; HCC, hepatocellular carcinoma; HR, hazard ratio; 95% CI, 95% confidence interval; CHC, chronic hepatitis C; CHB, chronic hepatitis B; NAFLD, nonalcoholic fatty liver disease.

**
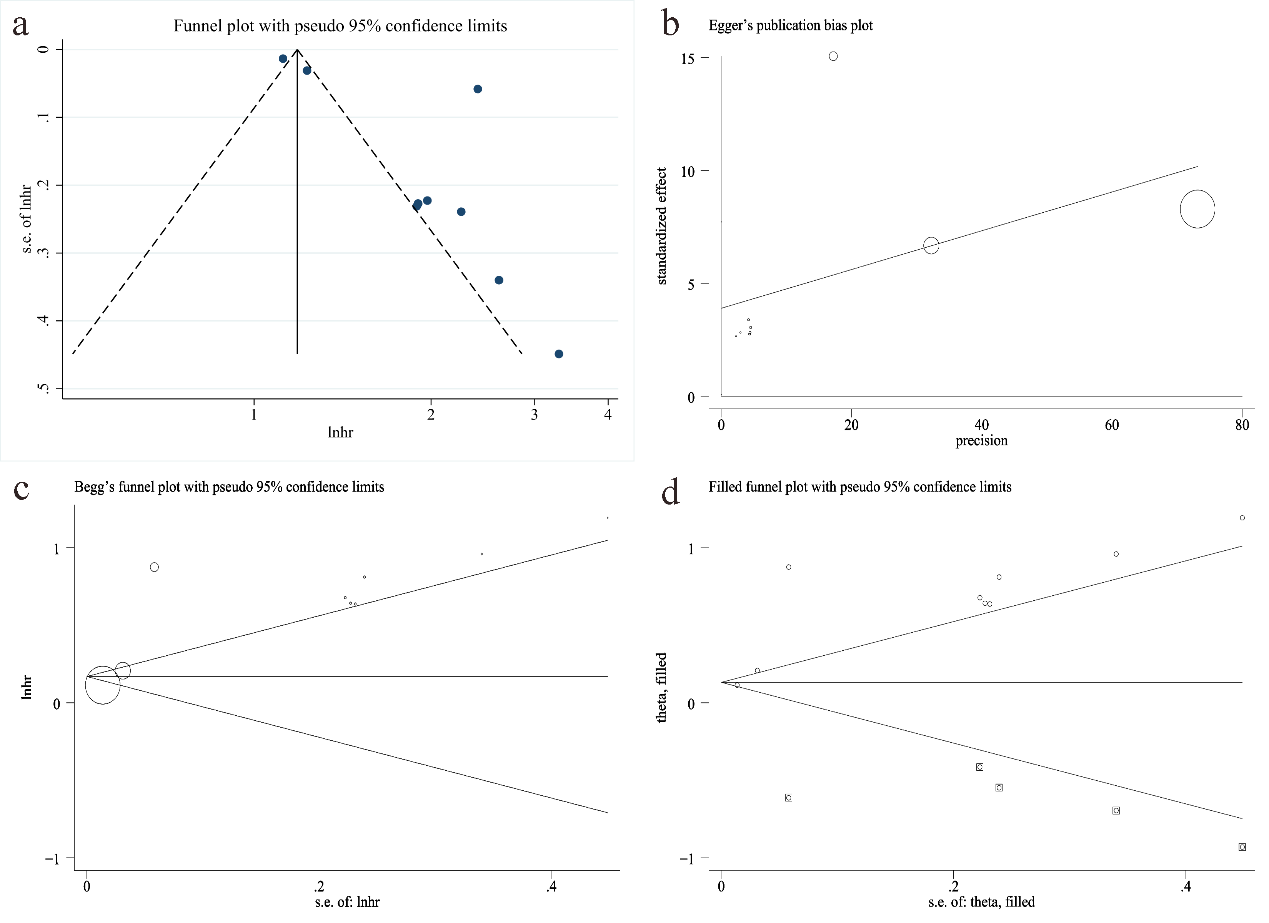
**

**Figure S2 publication bias of the association between MetS and HCC risk:** (a) funnel plot, (b) Egger's test, (c) Begg's test, (d) trim and fill method.


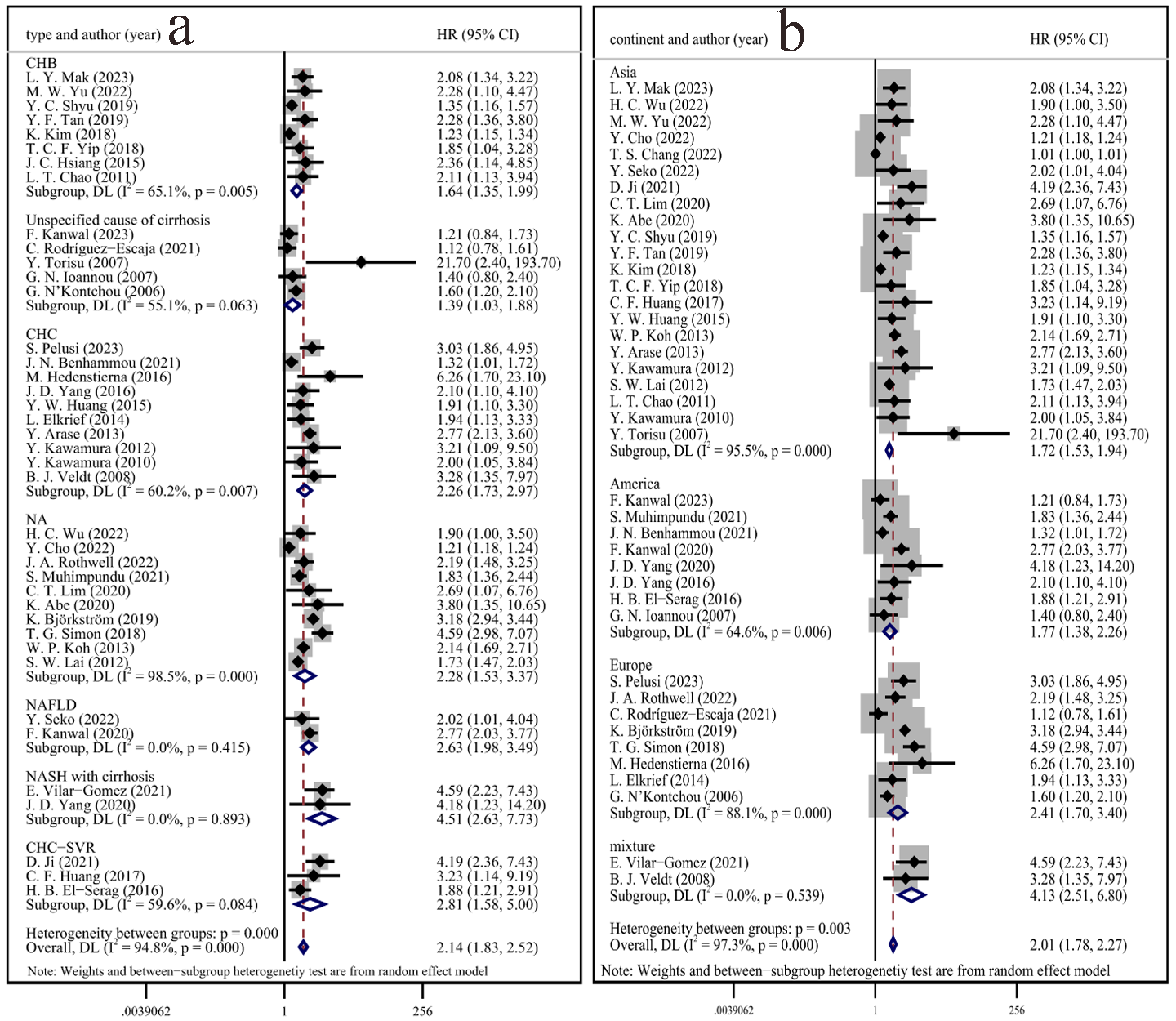


**Figure S3 Forest plots of subgroup showing the association between DM and HCC risk:** (a) cohort characteristics, (b) cohort source region. For the black diamond, its position represents the size of the HR studied, and the size of the square in grey surrounding the diamond represents the weight in the analysis. The length of the transverse line represents the corresponding 95% CI. Abbreviation: DM, diabetes mellitus; HCC, hepatocellular carcinoma; HR, hazard ratio; 95% CI, 95% confidence interval; CHC, chronic hepatitis C; CHB, chronic hepatitis B; NAFLD, nonalcoholic fatty liver disease; NASH, non-alcoholic steatohepatitis; SVR, sustained virologic response.


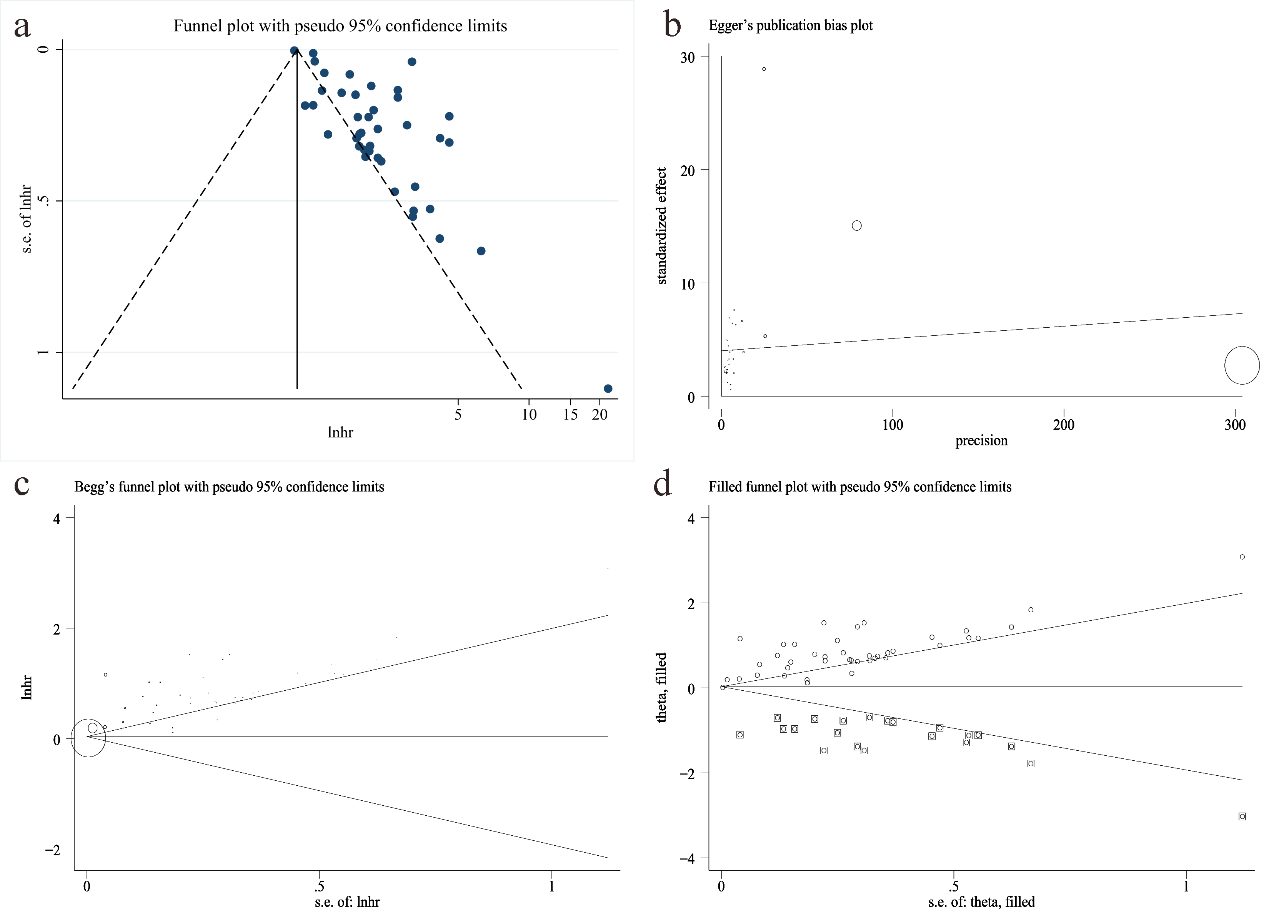


**Figure S4 publication bias of the association between DM and HCC risk:** (a) funnel plot, (b) Egger's test, (c) Begg's test, (d) trim and fill method.


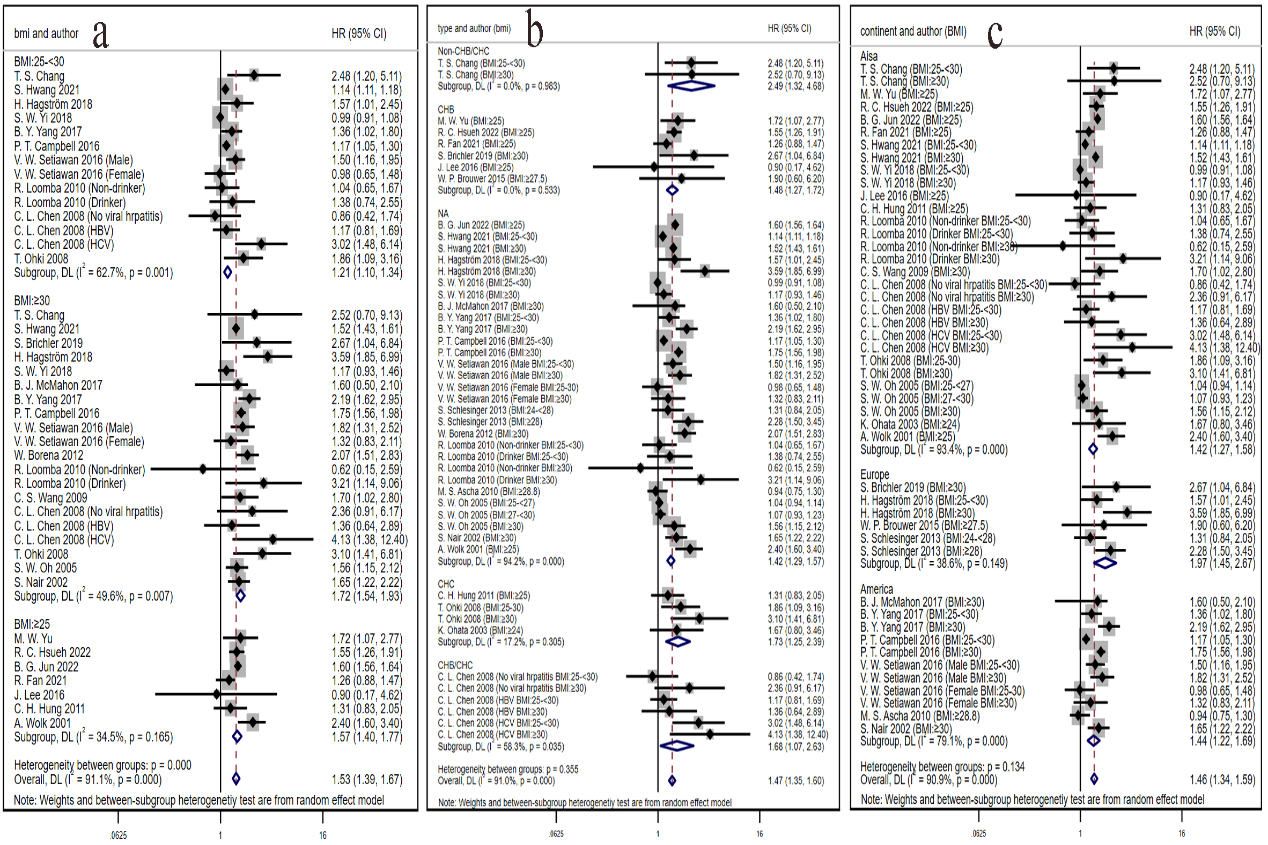
**Figure S5 Forest plots of subgroup showing the association between BMI and HCC risk:** (a)BMI range (kg/mg^2^), (b) cohort characteristics, (c) cohort source region. For the black diamond, its position represents the size of the HR studied, and the size of the square in grey surrounding the diamond represents the weight in the analysis. The length of the transverse line represents the corresponding 95% CI. Abbreviation: BMI, body mass index; HCC, hepatocellular carcinoma; HR, hazard ratio; 95% CI, 95% confidence interval; CHC, chronic hepatitis C; CHB, chronic hepatitis B.


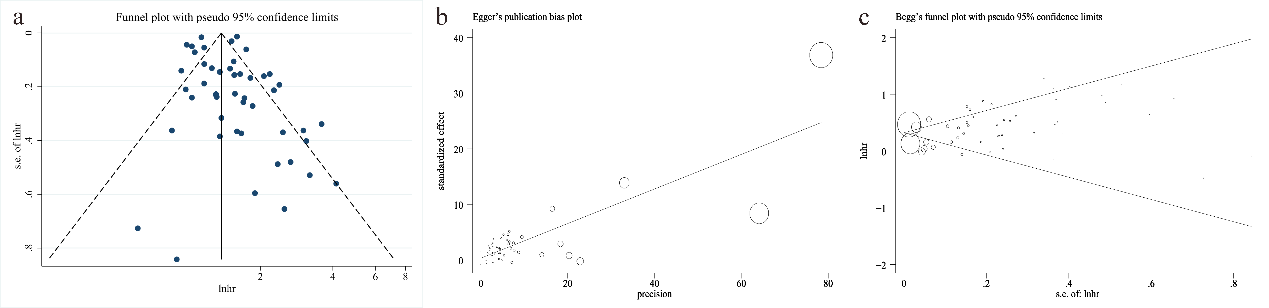


**Figure S6 publication bias of the association between BMI and HCC risk:** (a) funnel plot, (b) Egger's test, (c) Begg's test


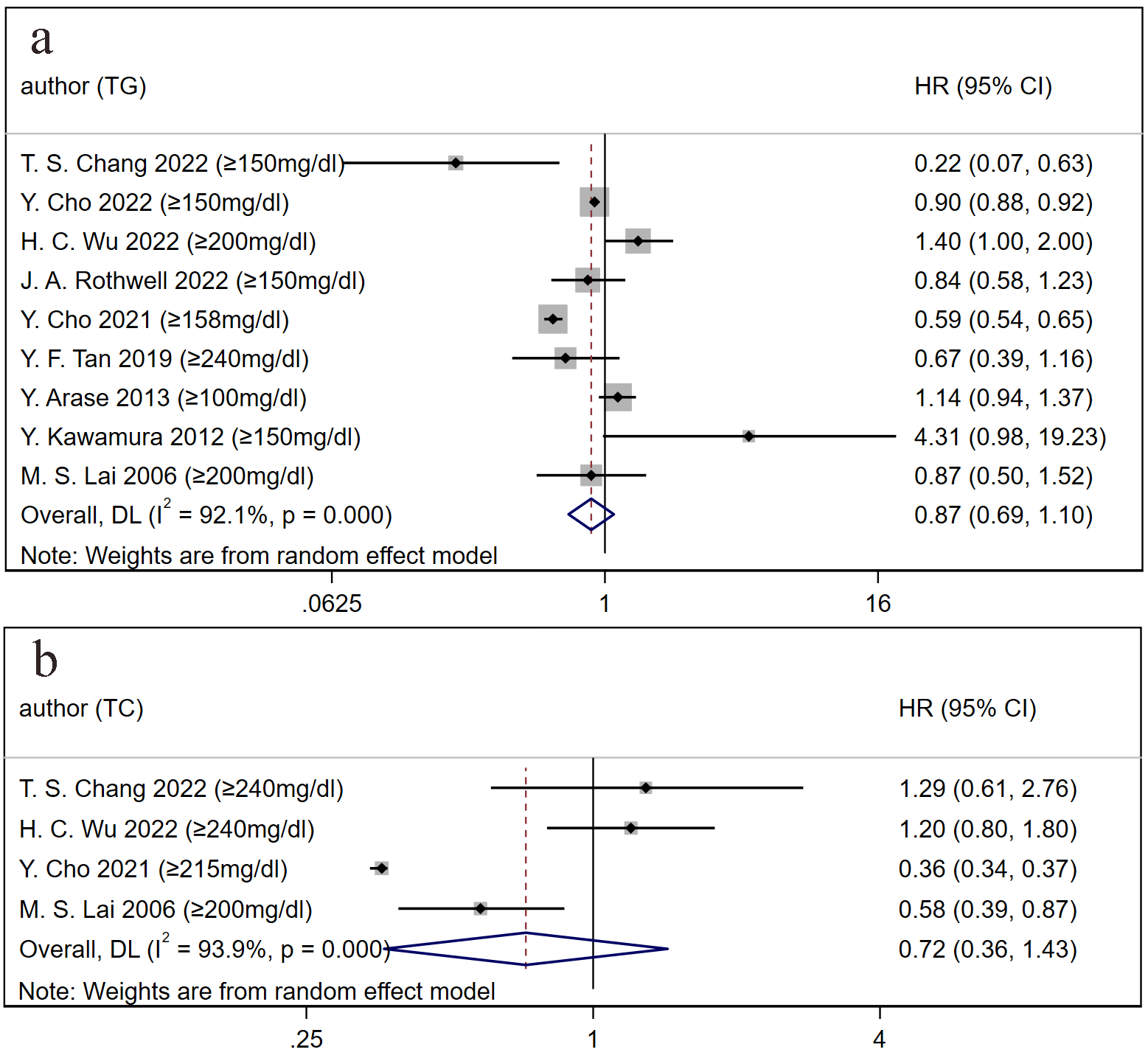


**Figure S7 Forest plots of all the studies showing the association between dyslipidemia and HCC:** (a) TG; (b) TC. For the diamond, its position represents the size of the HR studied, and the size of the square in grey surrounding the diamond represents the weight in the analysis. The length of the transverse line represents the corresponding 95% CI. Abbreviation: TG, total cholesterol; TC, triglyceride; HCC, hepatocellular carcinoma; HR, hazard ratio; 95% CI, 95% confidence interval.

**
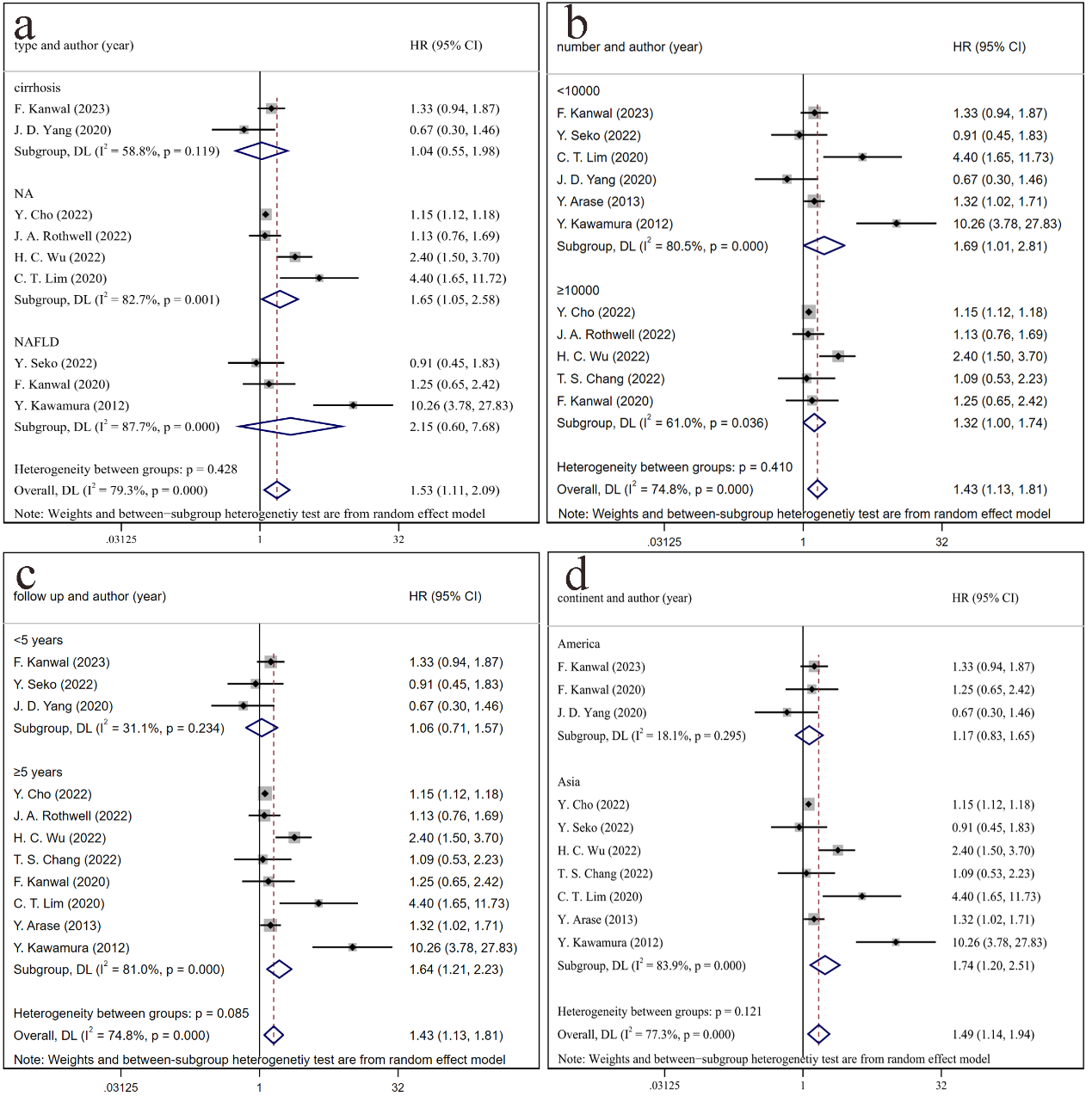
**

**Figure S8 Forest plots of subgroup showing the association between hypertension and HCC risk:** (a) cohort characteristics, (b) number of subjects, (c) media follow-up time, (d) cohort source region. For the black diamond, its position represents the size of the HR studied, and the size of the square in grey surrounding the diamond represents the weight in the analysis. The length of the transverse line represents the corresponding 95% CI. Abbreviation: HCC, hepatocellular carcinoma; HR, hazard ratio; 95% CI, 95% confidence interval; NAFLD, nonalcoholic fatty liver disease.


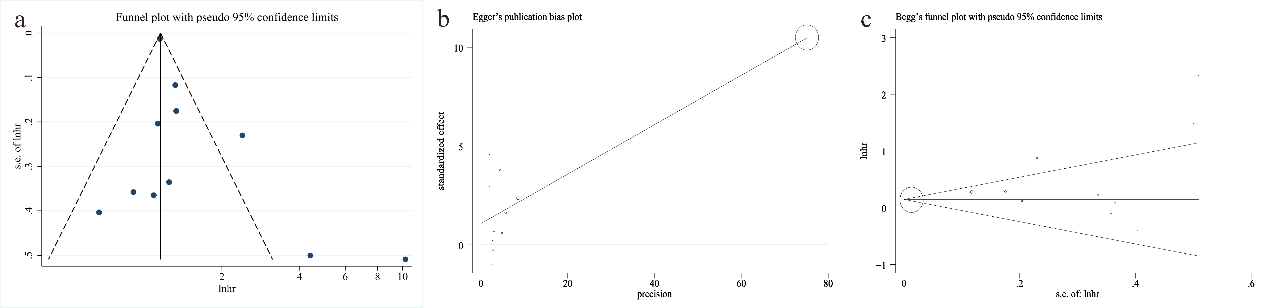


**Figure S9 publication bias of the association between hypertension and HCC risk:** (a) funnel plot, (b) Egger's test, (c) Begg's test.

**Supplementary Table S1: PRISMA Checklist**

| **Section and Topic** | **Item #** | **Checklist item** | **reported on** **section** |
| --- | --- | --- | --- |
| **TITLE** | | |  |
| Title | 1 | Identify the report as a systematic review. | Title page |
| **ABSTRACT** | | |  |
| Abstract | 2 | See the PRISMA 2020 for Abstracts checklist. | Title page |
| **INTRODUCTION** | | |  |
| Rationale | 3 | Describe the rationale for the review in the context of existing knowledge. | Introduction section |
| Objectives | 4 | Provide an explicit statement of the objective(s) or question(s) the review addresses. | Introduction section |
| **METHODS** | | |  |
| Eligibility criteria | 5 | Specify the inclusion and exclusion criteria for the review and how studies were grouped for the syntheses. | Methods section |
| Information sources | 6 | Specify all databases, registers, websites, organisations, reference lists and other sources searched or consulted to identify studies. Specify the date when each source was last searched or consulted. | Methods section |
| Search strategy | 7 | Present the full search strategies for all databases, registers and websites, including any filters and limits used. | Methods section |
| Selection process | 8 | Specify the methods used to decide whether a study met the inclusion criteria of the review, including how many reviewers screened each record and each report retrieved, whether they worked independently, and if applicable, details of automation tools used in the process. | Methods section |
| Data collection process | 9 | Specify the methods used to collect data from reports, including how many reviewers collected data from each report, whether they worked independently, any processes for obtaining or confirming data from study investigators, and if applicable, details of automation tools used in the process. | Methods section |
| Data items | 10a | List and define all outcomes for which data were sought. Specify whether all results that were compatible with each outcome domain in each study were sought (e.g. for all measures, time points, analyses), and if not, the methods used to decide which results to collect. | Methods section |
|  | 10b | List and define all other variables for which data were sought (e.g. participant and intervention characteristics, funding sources). Describe any assumptions made about any missing or unclear information. | Methods section |
| Study risk of bias assessment | 11 | Specify the methods used to assess risk of bias in the included studies, including details of the tool(s) used, how many reviewers assessed each study and whether they worked independently, and if applicable, details of automation tools used in the process. | Methods section |
| Effect measures | 12 | Specify for each outcome the effect measure(s) (e.g. risk ratio, mean difference) used in the synthesis or presentation of results. | Methods section |
| Synthesis methods | 13a | Describe the processes used to decide which studies were eligible for each synthesis (e.g. tabulating the study intervention characteristics and comparing against the planned groups for each synthesis (item #5)). | Methods section |
|  | 13b | Describe any methods required to prepare the data for presentation or synthesis, such as handling of missing summary statistics, or data conversions. | Methods section |
|  | 13c | Describe any methods used to tabulate or visually display results of individual studies and syntheses. | Methods section |
|  | 13d | Describe any methods used to synthesize results and provide a rationale for the choice(s). If meta-analysis was performed, describe the model(s), method(s) to identify the presence and extent of statistical heterogeneity, and software package(s) used. | Methods section |
|  | 13e | Describe any methods used to explore possible causes of heterogeneity among study results (e.g. subgroup analysis, meta-regression). | Methods section |
|  | 13f | Describe any sensitivity analyses conducted to assess robustness of the synthesized results. | Methods section |
| Reporting bias assessment | 14 | Describe any methods used to assess risk of bias due to missing results in a synthesis (arising from reporting biases). | Methods section |
| Certainty assessment | 15 | Describe any methods used to assess certainty (or confidence) in the body of evidence for an outcome. | Methods section |
| **RESULTS** | | |  |
| Study selection | 16a | Describe the results of the search and selection process, from the number of records identified in the search to the number of studies included in the review, ideally using a flow diagram. | Results section |
|  | 16b | Cite studies that might appear to meet the inclusion criteria, but which were excluded, and explain why they were excluded. | Results section |
| Study characteristics | 17 | Cite each included study and present its characteristics. | Results section |
| Risk of bias in studies | 18 | Present assessments of risk of bias for each included study. | Results section |
| Results of individual studies | 19 | For all outcomes, present, for each study: (a) summary statistics for each group (where appropriate) and (b) an effect estimate and its precision (e.g. confidence/credible interval), ideally using structured tables or plots. | Results section |
| Results of syntheses | 20a | For each synthesis, briefly summarise the characteristics and risk of bias among contributing studies. | Results section |
|  | 20b | Present results of all statistical syntheses conducted. If meta-analysis was done, present for each the summary estimate and its precision (e.g. confidence/credible interval) and measures of statistical heterogeneity. If comparing groups, describe the direction of the effect. | Results section |
|  | 20c | Present results of all investigations of possible causes of heterogeneity among study results. | Results section |
|  | 20d | Present results of all sensitivity analyses conducted to assess the robustness of the synthesized results. | Results section |
| Reporting biases | 21 | Present assessments of risk of bias due to missing results (arising from reporting biases) for each synthesis assessed. | Results section |
| Certainty of evidence | 22 | Present assessments of certainty (or confidence) in the body of evidence for each outcome assessed. | Results section |
| **DISCUSSION** | | |  |
| Discussion | 23a | Provide a general interpretation of the results in the context of other evidence. | Discussion section |
|  | 23b | Discuss any limitations of the evidence included in the review. | Discussion section |
|  | 23c | Discuss any limitations of the review processes used. | Discussion section |
|  | 23d | Discuss implications of the results for practice, policy, and future research. | Discussion section |
| **OTHER INFORMATION** | | |  |
| Registration and protocol | 24a | Provide registration information for the review, including register name and registration number, or state that the review was not registered. | Methods section |
|  | 24b | Indicate where the review protocol can be accessed, or state that a protocol was not prepared. | Methods section |
|  | 24c | Describe and explain any amendments to information provided at registration or in the protocol. | Methods section |
| Support | 25 | Describe sources of financial or non-financial support for the review, and the role of the funders or sponsors in the review. | Founding |
| Competing interests | 26 | Declare any competing interests of review authors. | Declarations |
| Availability of data, code and other materials | 27 | Report which of the following are publicly available and where they can be found: template data collection forms; data extracted from included studies; data used for all analyses; analytic code; any other materials used in the review. | Methods section |

**Supplementary Table S2: search strategy**

| **Search number** | **Query** |
| --- | --- |
| 1 | Glucose[Title/Abstract] OR 2h Glucose[Title/Abstract] OR Blood Glucose[Title/Abstract] OR Glycosuria[Title/Abstract] OR Hyperglycemia[Title/Abstract] OR Hypoglycemia[Title/Abstract] OR Blood Sugar[Title/Abstract] OR Sugar, Blood[Title/Abstract] OR Glucose, Blood[Title/Abstract] OR Fasting Glucose[Title/Abstract] OR HbA1c[Title/Abstract] OR blood HbA1c protein, human[Title/Abstract] OR HbA1c protein, human[Title/Abstract] OR Glycated Hemoglobin[Title/Abstract] OR Hemoglobin A1c[Title/Abstract] OR Fasting Insulin[Title/Abstract] OR "Diabetes Mellitus"[Mesh] OR Diabetes[Title/Abstract] AND epidemiologic studies[MeSH Terms] OR cohort studies[MeSH Terms] OR "epidemiologic"[Text Word] OR "cohort"[Text Word] OR "longitudinal"[Text Word] |
| 2 | "Hypertension"[MeSH Terms] OR blood pressure high[Title/Abstract] OR blood pressures high[Title/Abstract] OR high blood pressure[Title/Abstract] OR high blood pressures[Title/Abstract] |
| 3 | "Triglycerides"[Mesh] OR Triacylglycerol[Title/Abstract] OR Triacylglycerols[Title/Abstract] OR "Lipoproteins, HDL"[Mesh] OR HDL Lipoproteins[Title/Abstract] OR Heavy Lipoproteins[Title/Abstract] OR Lipoproteins, Heavy[Title/Abstract] OR High-Density Lipoproteins[Title/Abstract] OR High Density Lipoproteins[Title/Abstract] OR Lipoproteins, High-Density[Title/Abstract] OR alpha-Lipoproteins[Title/Abstract] OR alpha Lipoproteins[Title/Abstract] OR alpha-1 Lipoprotein[Title/Abstract] OR "Cholesterol, LDL"[Mesh] OR Low Density Lipoprotein Cholesterol[Title/Abstract] OR beta-Lipoprotein Cholesterol[Title/Abstract] OR Cholesterol, beta-Lipoprotein[Title/Abstract] OR beta Lipoprotein Cholesterol[Title/Abstract] OR LDL Cholesterol[Title/Abstract] OR Cholesteryl Linoleate, LDL[Title/Abstract] OR LDL Cholesteryl Linoleate[Title/Abstract] OR Total Cholesterol[Title/Abstract] OR Cholesterol[Title/Abstract] OR "Obesity"[Mesh] OR obese[Title/Abstract] OR adiposity[Title/Abstract] OR overweight[Title/Abstract] OR bodyweight[Title/Abstract] OR body mass index[Title/Abstract] OR body fat[Title/Abstract] OR body fat mass[Title/Abstract] |
| 4 | "Body Mass Index"[Mesh] OR "Obesity, Abdominal"[Mesh] OR Abdominal Obesities[Title/Abstract] OR Obesities, Abdominal[Title/Abstract] OR Central Obesity[Title/Abstract] OR Central Obesities[Title/Abstract] OR Obesities, Central[Title/Abstract] OR Obesity, Central[Title/Abstract] OR Abdominal Obesity[Title/Abstract] OR Obesity, Visceral[Title/Abstract] OR Visceral Obesity[Title/Abstract] OR Obesities, Visceral[Title/Abstract] OR Visceral Obesities[Title/Abstract] OR "Body Mass Index"[Mesh] OR Index, Body Mass[Title/Abstract] OR Quetelet Index[Title/Abstract] OR Index, Quetelet[Title/Abstract] OR Quetelet's Index[Title/Abstract] OR Quetelets Index[Title/Abstract]  "Waist Circumference"[Mesh] OR Circumference, Waist[Title/Abstract] OR Circumferences, Waist[Title/Abstract] OR Waist Circumferences[Title/Abstract] OR "Waist-Hip Ratio"[Mesh] OR Ratio, Waist-Hip[Title/Abstract] OR Ratios, Waist-Hip[Title/Abstract] OR Waist Hip Ratio[Title/Abstract] OR Waist-Hip Ratios[Title/Abstract] OR Waist-to-Hip Ratio[Title/Abstract] OR Ratio, Waist-to-Hip[Title/Abstract] OR Ratios, Waist-to-Hip[Title/Abstract] OR Waist to Hip Ratio[Title/Abstract] OR Waist-to-Hip Ratios[Title/Abstract] OR "Waist-Height Ratio"[Mesh] OR Ratio, Waist-Height[Title/Abstract] OR Ratios, Waist-Height[Title/Abstract] OR Waist Height Ratio[Title/Abstract] OR Waist-Height Ratios[Title/Abstract] OR Height-Weight Ratio[Title/Abstract] OR Height Weight Ratio[Title/Abstract] OR Height-Weight Ratios[Title/Abstract] OR Ratio, Height-Weight[Title/Abstract] OR Ratios, Height-Weight[Title/Abstract] OR Waist to Height Ratio[Title/Abstract] OR "hip Circumference"[Title/Abstract] OR Circumference, hip[Title/Abstract] OR Circumferences, hip[Title/Abstract] OR hip Circumferences[Title/Abstract] |
| 5 | epidemiologic studies [MeSH Terms] OR cohort studies[MeSH Terms] OR "epidemiologic"[Text Word] OR "cohort"[Text Word] OR "longitudinal"[Text Word] OR "follow up"[Text Word] OR observational[Text Word] OR prospective[Text Word] |
| 6 | "Carcinoma, Hepatocellular"[Mesh] OR Liver Neoplasms[Title/Abstract] OR hepatic neoplasm[Title/Abstract] OR hepatocellular cancer[Title/Abstract] OR hepatic cancer[Title/Abstract] OR liver cancer[Title/Abstract] OR liver cell carcinoma[Title/Abstract] OR liver tumor[Title/Abstract] OR hepatocellular carcinoma[Title/Abstract] OR HCC[Title/Abstract] |
| 7 | 1 AND 5 AND 6 |
| 8 | 2 AND 5 AND 6 |
| 9 | 3 AND 5 AND 6 |
| 10 | 4 AND 5 AND 6 |

2\

**Supplementary Table S3: Quality assessment for cohort studies by checklist (based on Newcastle-Ottawa Scale)**

| **Author** | **Year** | **Selection (4 points)** | **Comparability (2 points)** | **Outcome (3 points)** | **Score (up to 9 points)** |
| --- | --- | --- | --- | --- | --- |
| S. Pelusi | 2023 | 4 | 2 | 2 | 8 |
| L. Y. Mak | 2023 | 3 | 1 | 3 | 7 |
| F. Kanwal | 2023 | 3 | 1 | 2 | 6 |
| T. S. Chang | 2022 | 4 | 1 | 2 | 8 |
| M. W. Yu | 2022 | 4 | 2 | 3 | 9 |
| Y. Cho | 2022 | 3 | 1 | 2 | 8 |
| R. C. Hsueh | 2022 | 3 | 1 | 3 | 8 |
| Y. Seko | 2022 | 4 | 2 | 3 | 9 |
| B. G. Jun | 2022 | 4 | 1 | 2 | 8 |
| H. C. Wu | 2022 | 3 | 1 | 2 | 7 |
| J. A. Rothwell | 2022 | 3 | 2 | 2 | 7 |
| Y. G. Chen | 2022 | 4 | 2 | 3 | 9 |
| S. Muhimpundu | 2021 | 4 | 2 | 3 | 9 |
| Y. Cho | 2021 | 3 | 2 | 3 | 8 |
| R. Fan | 2021 | 3 | 1 | 2 | 6 |
| D. Ji | 2021 | 3 | 2 | 2 | 7 |
| E. Vilar-Gomez | 2021 | 3 | 2 | 3 | 8 |
| J. N. Benhammou | 2021 | 3 | 2 | 3 | 8 |
| S. Hwang | 2021 | 3 | 2 | 3 | 8 |
| Y. B. Lee | 2021 | 4 | 1 | 3 | 8 |
| C. Rodríguez-Escaja | 2021 | 4 | 2 | 3 | 9 |
| K. Abe | 2020 | 3 | 2 | 2 | 7 |
| C. T. Lim | 2020 | 3 | 1 | 2 | 6 |
| A. A. Florio | 2020 | 4 | 2 | 3 | 9 |
| F. Kanwal | 2020 | 4 | 2 | 2 | 8 |
| J. D. Yang | 2020 | 3 | 2 | 2 | 7 |
| Y. F. Tan | 2019 | 3 | 1 | 2 | 6 |
| Y. C. Shyu | 2019 | 4 | 2 | 2 | 8 |
| S. Brichler | 2019 | 4 | 1 | 2 | 7 |
| K. Björkström | 2019 | 3 | 2 | 3 | 8 |
| K. Kim | 2018 | 4 | 2 | 2 | 8 |
| H. Hagström | 2018 | 4 | 2 | 3 | 9 |
| A. Wong | 2018 | 3 | 2 | 3 | 8 |
| T. C. F. Yip | 2018 | 3 | 1 | 3 | 7 |
| T. G. Simon | 2018 | 3 | 2 | 3 | 8 |
| S. W. Yi | 2018 | 4 | 2 | 3 | 9 |
| C. F. Huang | 2017 | 4 | 1 | 3 | 8 |
| M. W. Yu | 2017 | 4 | 2 | 2 | 8 |
| B. J. McMahon | 2017 | 3 | 2 | 2 | 7 |
| B. Y. Yang | 2017 | 4 | 2 | 3 | 9 |
| J. Lee | 2016 | 3 | 2 | 2 | 8 |
| P. T. Campbell | 2016 | 2 | 2 | 3 | 7 |
| M. Hedenstierna | 2016 | 3 | 2 | 3 | 8 |
| J. D. Yang | 2016 | 3 | 2 | 1 | 6 |
| V. W. Setiawan | 2016 | 3 | 2 | 2 | 7 |
| H. B. El-Serag | 2016 | 3 | 2 | 3 | 8 |
| W. P. Brouwer | 2015 | 4 | 2 | 3 | 9 |
| J. C. Hsiang | 2015 | 2 | 3 | 3 | 7 |
| Y. W. Huang | 2015 | 3 | 2 | 3 | 8 |
| L. Elkrief | 2014 | 3 | 2 | 1 | 6 |
| W. P. Koh | 2013 | 4 | 2 | 3 | 9 |
| S. Schlesinger | 2013 | 4 | 2 | 2 | 8 |
| Y. Arase | 2013 | 3 | 1 | 3 | 7 |
| S. W. Lai | 2012 | 3 | 2 | 3 | 8 |
| Y. Kawamura | 2012 | 2 | 2 | 2 | 6 |
| W. Borena | 2012 | 4 | 2 | 3 | 9 |
| L. T. Chao | 2011 | 3 | 2 | 2 | 7 |
| C. H. Hung | 2011 | 4 | 1 | 3 | 8 |
| R. Loomba | 2010 | 4 | 2 | 2 | 8 |
| Y. Kawamura | 2010 | 4 | 2 | 2 | 8 |
| M. S. Ascha | 2010 | 3 | 2 | 2 | 7 |
| C. S. Wang | 2009 | 4 | 1 | 3 | 8 |
| C. L. Chen | 2008 | 3 | 2 | 1 | 6 |
| T. Ohki | 2008 | 4 | 2 | 2 | 8 |
| B. J. Veldt | 2008 | 4 | 2 | 2 | 8 |
| Y. Torisu | 2007 | 3 | 2 | 1 | 6 |
| G. N. Ioannou | 2007 | 4 | 2 | 3 | 9 |
| G. N'Kontchou | 2006 | 3 | 2 | 3 | 8 |
| M. S. Lai | 2006 | 3 | 2 | 3 | 8 |
| S. W. Oh | 2005 | 3 | 2 | 3 | 8 |
| K. Ohata | 2003 | 3 | 2 | 3 | 8 |
| S. Nair | 2002 | 4 | 2 | 3 | 9 |
| A. Wolk | 2001 | 3 | 2 | 2 | 7 |

**Supplementary Table S4:** **Publication bias of obesity and dyslipidemia and HCC risk**

| **Dyslipidemia** | **Is the funnel plot symmetrical?** | **Begg’s (p-value)** | **Egger’s (p-value)** |
| --- | --- | --- | --- |
| WC | Yes | 0.921 | 0.050 |
| WHR | Yes | 0.734 | 0.124 |
| WHtR | Yes | 1.000 | 0.450 |
| High TG | Yes | 0.348 | 0.741 |
| High TC | Yes | 0.308 | 0.070 |
| Low TC | Yes | 1.000 | NA |
| High HDL-C | Yes | 1.000 | 0.818 |
| Low HDL-C | Yes | 0.308 | 0.166 |
| High LDL-C | Yes | 1.000 | 0.933 |

Abbreviation: WC, waist circumference; WHR, waist-to-hip ratio; WHtR, waist-to-height ratio; TG, total cholesterol; TC, triglyceride; HDL-C, high-density lipoprotein cholesterol; LDL-C, low-density lipoprotein cholesterol.
